# ABE9 fused to SpRY Cas9 nickase enables precise generation of bystander free mouse models

**DOI:** 10.1038/s41598-026-40642-z

**Published:** 2026-02-20

**Authors:** Jun Kai Ong, Sayari Bhunia, Beate Hilbert, Vanessa Kirschner, Sascha Duglosz, Frank Zimmermann, Marc Freichel, Alex Cornean

**Affiliations:** 1 Institute of Pharmacology, Heidelberg University, 69120 Heidelberg, Germany; 2 DZHK (German Center for Cardiovascular Research), Partner Site Heidelberg/Mannheim, Heidelberg University, 69120 Heidelberg, Germany; 3 Heidelberg Biosciences International Graduate School (HBIGS), 69120 Heidelberg, Germany; 4 Interfacultary Biomedical Faculty (IBF), Heidelberg University, 69120 Heidelberg, Germany; 5 Present Address: Medizinische Klinik II, Uniklinikum Würzburg, 97078 Würzburg, Germany

**Keywords:** Adenine base editing, Precision editing, Mouse modelling, CRISPR-Cas9, hiPS cells, Biological techniques, Biotechnology, Developmental biology, Genetics

## Abstract

Point mutations cause many genetic disorders, but modelling them in organisms is technically challenging. Creating mouse models that mimic these mutations is crucial for establishing a causal relationship between mutations and disease phenotype, thereby supporting the development of therapeutic strategies. Adenine base editors (ABEs) can correct single-nucleotide variants (SNVs) in disease modelling without double-stranded breaks (DSBs) or donor DNA, achieving higher product purity than traditional Cas9 methods. Earlier ABE techniques faced issues like limited targetability, bystander editing, and off-target effects. By combining two editor advancements, we introduced and tested ABE9-SpRY, an improved ABE variant fused with a PAM-flexible SpRY-Cas9 nickase. Our results show that ABE9-SpRY effectively generates three out of four targeted A-to-G mutations in mouse embryos, achieving desired editing efficiencies of up to 96% in individual adult founder mice. Furthermore, we observe fewer off-target events at predicted DNA sites in mouse embryos and in an orthogonal R-loop assay compared with ABE8e-SpRY. ABE9-SpRY also enhances product purity in mouse embryos under pooled sgRNA injections and, as a proof-of-concept, at a single endogenous locus in human induced pluripotent stem cells (hiPSCs), relative to ABE8e-SpRY. Our findings support ABE9-SpRY’s precision at the loci tested and PAM-flexible versatility. Although performance remains sequence-dependent, these data support ABE9-SpRY as a PAM-flexible tool for generating precise point-mutation models where bystander editing is a concern.

**Supplementary Information:**

The online version contains supplementary material available at 10.1038/s41598-026-40642-z.

## Introduction

Disease models have been indispensable in advancing biomedical research by enabling the study of complex physiological and biological interactions that are difficult to replicate *in vitro*^1^. The mouse has emerged as the dominating biomedical model due to its genetic, biological, and behavioural similarities to humans, along with the availability of advanced genetic tools for manipulation^2^. Moreover, mouse models with disease-relevant mutations or mutations in a given drug receptor have tremendous value for the development and validation of the mode of action of new therapies^3–5^. In parallel, hiPSCs have also become a powerful *in vitro* model, providing a human-specific system for studying disease mechanisms, drug responses, and genetic modifications in a physiologically relevant context^6^.

The recent development of precise CRISPR-Cas-based gene editing tools has significantly expanded the possibilities for creating genetically engineered mouse models. Notably, the CRISPR-Cas9 system has enabled targeted genome modification by directing nuclease activity to specific genomic loci^7^. Its efficiency, widespread adaptation and simplicity have made CRISPR-Cas9 the most widely used system for gene editing. However, the CRISPR-Cas9 system has its limitations. Conventional Cas9 nuclease editing depends on creating a targeted DSB, which is mainly repaired by end-joining pathways and often results in insertions or deletions (indels) at the cut site^8^. Additionally, imperfect guide RNA–DNA complementarity can cause off-target binding and cleavage at similar genomic sites, leading to unintended mutations elsewhere^9^. Likewise, larger deletions^10,11^ and chromosomal aberrations^12–14^ have been reported to occur at significantly higher frequencies with DSB-inducing gene editing approaches.

To address the limitations of DSBs, DNA base editing has emerged, catalysed by a new class of tools known as base editors^15,16^. Base editors allow single-nucleotide conversions without requiring DSBs or donor DNA templates. ABEs are of particular interest for introducing A-to-G (or T-to-C) base transitions^15^, which are relevant to a wide range of genetic disorders. For example, G•C to A•T mutations account for approximately 47% of the most common pathogenic SNPs in the ClinVar database^17^. By fusing a Cas9 nickase (Cas9n) with a deaminase enzyme that converts adenine to inosine, then read as guanine by the cell, ABEs have been used to install point mutations with high efficiency and precision^15,18^.

**Fig. 1:**
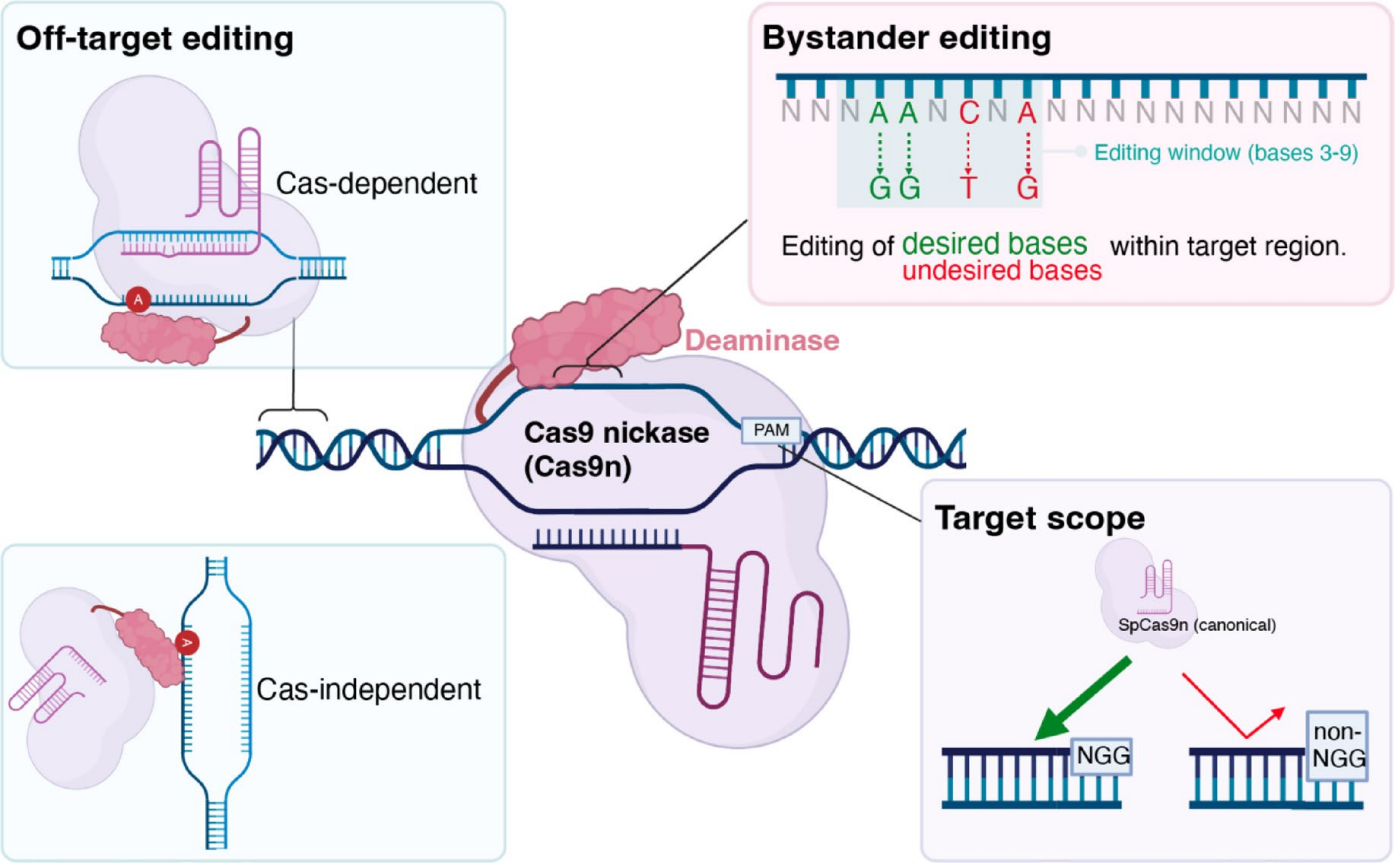
Limitations of disease modelling with ABEs. Schematic of limitations of ABEs in generating disease models. ABEs can exhibit Cas-dependent or -independent off-target editing. Besides targeted adenines (green), ABEs also have bystander activity on non-target bases (red) within the editing window (cyan box). SpCas9 employed in ABEs recognises a 3’ NGG PAM but does not bind optimally, or at all, to non-NGG PAM sites. This figure was partially prepared with BioRender.

ABEs therefore hold immense promise for enabling rapid, precise generation of disease models in animal and cell systems, especially in mice. However, current ABE approaches in mice are limited by bystander editing, issues of targetability and off-target (OT) editing (Fig. 1). Bystander editing occurs when nucleotides within the deaminase’s editing window, other than the target site(s), are also modified^17^, causing unwanted mutations and complicating the study of disease-specific mutations. Canonical ABEs are also limited by their reliance on the NGG protospacer adjacent motif (PAM) recognised by the commonly used *Streptococcus pyogenes* Cas9 nickase, which restricts the range of targetable sites in the genome^15^. Both Cas-dependent, guided by the sgRNAs’ base pairing properties to similar genomic regions^19,20^, and Cas9-independent off-target editing, based on deaminase activity on adenines in single-stranded DNA regions outside the targeted site^21,22^, have raised serious concerns. These limitations have driven efforts to improve ABEs through directed evolution and rational design. Next-generation variants, such as ABE8e^23^, exhibit enhanced deaminase activity, whereas ABE9 has increased specificity due to a narrower editing window^24^. Additionally, engineered Cas9 variants like SpRY, which have relaxed PAM requirements, have expanded the range of targetable sequences, allowing more flexible editing across the genome^25^. We therefore aimed to combine the specificity of the ABE9 deaminase with the flexibility of SpRY, thereby overcoming all three challenges and benchmarking these against ABE8e-SpRY^26^, to establish four different mouse models with precise point mutations in the ion channels TPC1, TPC2, and TRPM4. These channels play critical roles in physiological processes, and mutations in these channels have been linked to diseases, particularly affecting hepatic and cardiovascular tissues^27–31^.

To streamline the generation of disease models in mice and substantially reduce the number of required animals, we established a workflow to characterise and utilise ABE9-SpRY for the rapid introduction of desired mutations. After initial benchmarking and strategy design, we cloned and tested the desired sgRNA/ABE-SpRY combinations in mouse Neuro-2a (N2a) cells, a well-established, readily transfectable *in vitro* screening platform, followed by pooled injection of synthetic sgRNAs and SpRY-ABEs mRNA into mouse zygotes and analysis of all target and selected OT sites in E14 embryos. Our strategy enabled generating two mouse models carrying point mutations with minimal bystander editing and high editing efficiency for *Tpc1*^*I486T*^ and *Trpm4*^*L903P*^, which, due to its broad targetability, can be used to create a substantial fraction of A-to-G-based mouse models. We further tested whether the high-on-target purity of ABE9-SpRY in the mouse system also translates in human cells, by using hiPSCs as a validation platform. We achieved increased editing with undetectable bystander activity at the *TPC1*^*I485T*^ locus, suggesting a broader applicability of ABE9-SpRY.

## Results

### Efficient and precise A-to-G base editing with ABE9 in human cells

The most widely used ABE variant today is ABE8e, recognised for its high deaminase activity and processivity^23^. However, the increased catalytic activity coincides with a significant broadening of the editing window, expanding from adenine positions A4-A8 in ABEmax to A3-A10 in ABE8e^32^, as well as higher levels of off-target DNA and RNA editing^23,24,32^. Therefore, this rise in unwanted on-target bystander and off-target editing raises concerns regarding the safety of ABE8e for potential clinical applications^32^. To address these safety concerns, several approaches have frequently been employed in the past (Table 1): introducing structure-guided or evolved mutations in the deaminase domain^18,23,24,32–36^, splitting the editor^37,38^, using low mismatch tolerance Cas variants^39^, and fusing the ABE with an effector domain^40^.

**Table 1:** Overview of available approaches to overcome precision, off-target (OT) and targetability issues.

Goal	Type	Editor	Efficiency*	Editing window (vs 3–10*)	Off-targets*	References
Precision	Deaminase mutations	ABE9e-NG (R111T, N127K, Q154R) ABE8e-N108Q **ABE9 (N108Q, L145T)**	Similar Similar ** > -20%**	(3)4–7(8) at similar activity 4–7 **5–6**	**Reduced DNA and RNA OTs**	^32^ ^24^
	Split editor	PIGS-ABE8e sABE v3.22	> -40% > -25%	4–9 4–7	Reduced DNA and RNA OTs	^37^ ^38^
	Effector domain	ABE8e-e18	> -10%	5–7	Reduced DNA OTs	^40^
Off-target reduction	Deaminase mutations	ABE8e-V106W ABE8.17-m-V106W ABE8e-WA ABE8e-WQ	Similar > -10% Similar > -20%	Similar Similar 4–8 4–8	Reduction of DNA & RNA OTs Reduced RNA OTs	^23^ ^18^ ^33^
	Deaminase mutation (+ Cas9 variant)	SpRY-ABE8e-F148A CE-ABE8e-SpRY CE-ABE8e-dV-SpRY	Similar Similar Similar	Similar Similar Similar	Reduced RNA OTs Slightly reduced DNA OTs and reduced RNA OTs Reduced indels, RNA and DNA OTs	^36^ ^34^ ^35^
	Cas9 variant	Sniper2L	Low mismatch tolerance (only 1 tolerated)			^39^
Target-ability	Cas9 variant	**ABEmax-SpRY** ABE8e-SpRYc	**PAMless; targetability across NNN PAMs but likely massive increase in DNA OTs** PAMless; at certain selected loci more unbiased than ABE8e-SpRY			^25^ ^47^

*Mostly compared to ABE8e.

Among all these options, ABE9, which was derived from ABE8e by introducing two point mutations N108Q and L145T in the deaminase domain, appeared to be the most suitable due to its narrowest editing window to date, located between protospacer positions 5–6, and its strong properties for reducing off-target effects in DNA and RNA^24^. To this end, we compared ABE8e and ABE9 at four previously reported ABE target sites in HEK293T cells to confirm their properties (Supplementary Fig. 1). Indeed, the editing window of ABE9 was specific to positions A5 and A6, with some minimal activity at A4, whereas ABE8e was highly active between A3 and A8 with an extended activity shoulder to A13. Overall, the average activity at A5 was reduced from 43.6 ± 5.9% (mean ± s.d.%) in ABE8e to 34.5 ± 8.8% in ABE9, corresponding to an average reduction of 21% in editing activity, in line with previous work^24^ (Supplementary Fig. 1a–c). Interestingly, indel frequencies in HEK293T cells were locus-dependent: ABE9 showed higher indel rates than ABE8e at two of the four benchmark sites, while exhibiting reduced bystander C-to-T editing at three sites and achieving greater overall precision across all four sites (Supplementary Fig. 1d–f). Given that our primary goal is to generate precise point-mutation models with minimal bystander outcomes, we prioritised this improved precision over maximal bulk editing efficiency and therefore selected ABE9 for subsequent testing at our loci of interest.

### ABE9-SpRY shows higher product purity in a mouse neuroblastoma line (N2a) at four disease-relevant loci

**Fig. 2:**
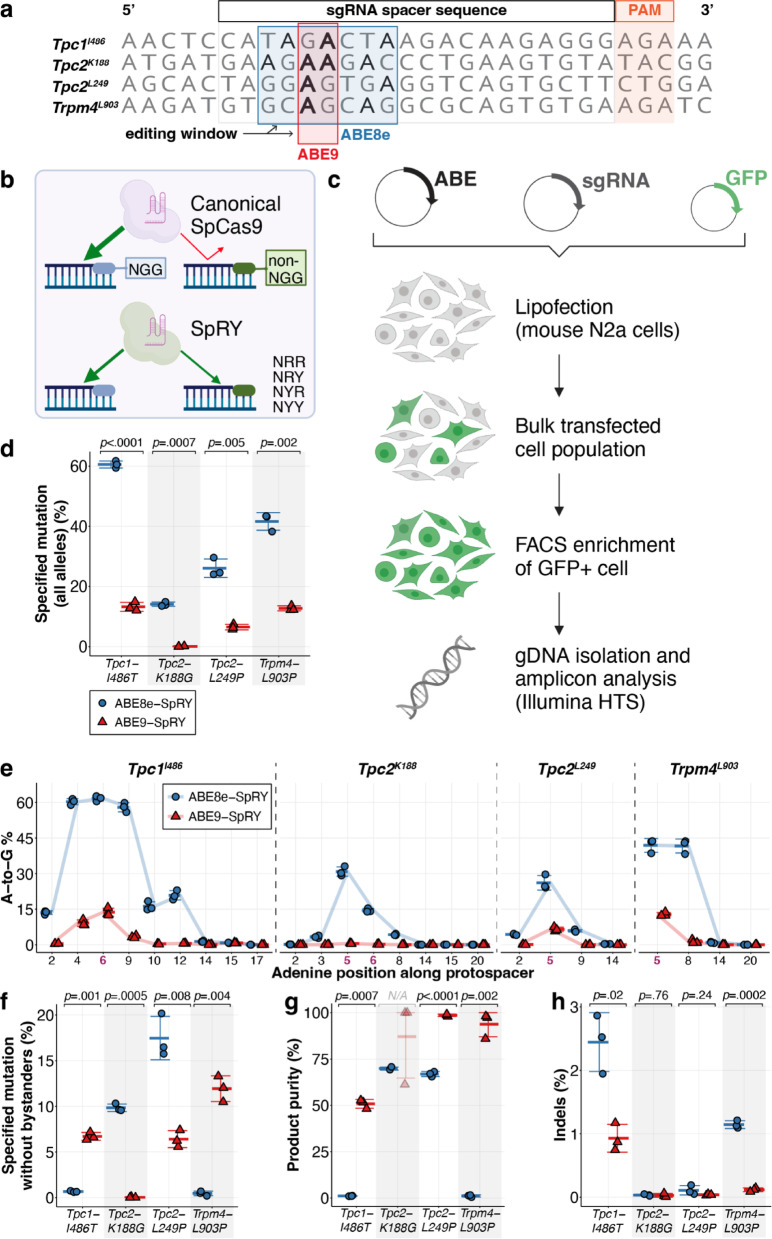
Evaluation of ABE-SpRY constructs in target sites of interest in mouse N2a cells. (**a**) Design of sgRNAs for optimal adenine base editing. Target adenines (bolded in red) for each locus are positioned within the optimal editing windows of ABE8e and ABE9 deaminases with the corresponding PAM sites shown. (**b**) The Cas9 variant SpRY can target non-NGG PAM sites, unlike the wild-type SpCas9. (**c**) N2a cells were co-transfected with three plasmids: the ABE plasmid, sgRNA plasmid, and GFP plasmid. GFP-positive cells were sorted, and genomic DNA (gDNA) was extracted for subsequent targeted sequencing analysis. (**d**) Maximum editing efficiency observed for the target adenine with ABE8e- or ABE9-SpRY. The “specified mutation (all alleles)” includes all reads that carry the intended substitution, regardless of bystander edits within the analysed window. (**e**) A-to-G editing efficiencies of ABE8e and ABE9-SpRY at each adenine position along the protospacer region for the four target sites. The positions of adenines of interest are bolded in magenta. (**f**) Editing efficiency of the target adenine using ABE8e- or ABE9-SpRY. The “specified mutation without bystanders” reports only clean desired-only (“perfect”) alleles within the analysed window. (**g**) Product purity was calculated as the percentage of reads carrying the desired substitution that were free of bystander edits within the quantification window: 100 × (desired-only / (desired-only + "desired + bystander")). (**h**) Frequency of indels at each of the four target sites. Individual data points for three independent biological replicates are shown. Error bars indicate standard deviation (SD); bounds were capped at 0% and 100% where the mean ± SD exceeded the valid range. Parts of the schematics in (**b**) and (**c**) were created with BioRender.com.

We first designed the sgRNAs for the four loci to position the target adenines within the optimal editing windows of the deaminases of ABE8e and ABE9, respectively (Fig. 2a). However, the canonical architectures of PAM-interacting motifs in both ABEs, when fused to the standard SpCas9n, proved unsuitable due to the lack of the NGG PAM sequence.

To address this limitation, we next considered alternative Cas9 variants that have different or relaxed PAM requirements, including SaCas9, xCas9, SpCas9-NG, SpCas9-VQR, and SpRY^25,41–46^. Instead of an “on-demand” context-dependent approach, we developed a versatile editing strategy that can be applied to all four loci of interest by utilising a single construct and the appropriate sgRNA. SpRY^25^ and SpRYc^47^ have emerged as potentially universal Cas9 variants with PAMless targetability across NNN PAMs (Table 1). SpRY stands out for having the broadest and most relaxed PAM compatibility and has previously been tested with ABE8e variants^34–36^. We fused a SpRY nickase (in the following simply “SpRY”, bears these substitutions compared to SpCas9 nickase: A61R/L1111R/D1135L/S1136W/G1218K/E1219Q/N1317R/A1322R/R1333P/R1335Q/T1337R) to the TadA domains of ABE8e or ABE9 to streamline the experimental workflow and maximise the potential for successful genome editing outcomes (Fig. 2b). These SpRY base editors can, in theory, effectively target spacers with NRN (G or A) PAMs, and to a lesser degree, NYN (T or C) PAMs^25^. Since the compatibility of deaminase domains with Cas9 variants is not universal, and their efficiency and editing activity can vary with the Cas9 scaffold used^18,48,49^, an initial evaluation was necessary. We therefore compared ABE9-SpRY with ABE8e-SpRY in N2a cells using plasmid lipofection with the respective sgRNAs at four loci under identical transfection and enrichment conditions (Fig. 2c; Methods).

Deep sequencing of transfected N2a cells revealed that the desired editing occurred at all four targets for ABE8e-SpRY, ranging from 14.1 ± 0.7% to 60.6 ± 1.2%. In contrast, only three targets showed targeted A-to-G editing with ABE9-SpRY, ranging from 6.5 ± 0.9% to 13.2 ± 1.5% across all tested sites (Fig. 2d). While ABE8e-SpRY displayed modest editing of 14.1 ± 0.7% at the *Tpc2-K188* locus, no desired editing was noted with ABE9-SpRY. A closer examination of adenine editing profiles across the protospacers confirmed a consistent trend across all four loci: ABE8e-SpRY exhibited a broad activity window across multiple adenines (with locus-dependent shoulders extending to more distal positions), whereas ABE9-SpRY showed a more confined editing profile with markedly reduced bystander activity (Fig. 2e). For example, at the *Tpc1-I486* locus, ABE8e-SpRY editing extended to distal positions (13.5 ± 0.5% at A2 and 20.8 ± 0.6% at A12), whereas ABE9-SpRY activity was largely restricted to a narrower region. The highest ABE8e-SpRY activities were observed at A5 (*Tpc2-K188*, *Tpc2-L249*, *Trpm4-L903*) and A6 (*Tpc1-I486)*, with 30.8 ± 0.5%, 26.1 ± 6.5%, 41.9 ± 12.8%, and 61.6 ± 12.7% A-to-G editing, respectively. By contrast, ABE9-SpRY demonstrated the enhanced precision previously observed for ABE9, showing a narrower activity window with reduced conversion at flanking adenines across loci; at the *Tpc1-I486* locus, activity spanned from A4 to A9, with 9.1 ± 1.3% and 3.3 ± 0.5% editing at A4 and A9, respectively. We also measured the highest efficiencies for ABE9-SpRY at A5 and A6, respectively, with 6.5 ± 0.9%, 12.8 ± 0.8%, and 13.7 ± 1.7% efficiency for *Tpc2-L249*, *Trpm4-L903*, and *Tpc1-I486,* respectively. In N2a cells at four disease-relevant loci, this narrower editing window of ABE9-SpRY also led to a larger fraction of edited alleles containing no bystander mutations compared to ABE8e-SpRY (Fig. 2f), as indicated by the substantially higher product purity, ranging from 50.9 ± 2.4% to 98.6 ± 0.6%, compared to 1.1 ± 0.1% to 69 ± 0.8% for ABE8e-SpRY (Fig. 2g). Product purity calculations were restricted to our predefined CRISPResso2 quantification window (spacer − 7 nt to spacer + PAM (+ 3 nt); Methods). Furthermore, as ABE9-SpRY showed lower overall A-to-G conversion at these loci (Fig. 2d–e), differences in product purity may reflect both window narrowing and reduced activity. Notably, the desired *Tpc1*^*I486T*^ and *Trpm4*^*L903P*^ mutations could not be introduced without bystanders using ABE8e-SpRY (0.7 ± 0.1% and 0.5 ± 0.3%), whereas ABE9-SpRY achieved 6.7 ± 0.4% and 11.9 ± 1.4% precise editing at these sites, respectively. Lastly, ABE9-SpRY demonstrated reduced indel frequencies compared to ABE8e-SpRY across all four sites, peaking at 0.9 ± 0.2% (*Tpc1-I486*) (Fig. 2h). Since these *in vitro* data were obtained from a single mouse neuroblastoma cell line and four loci, further testing is needed to determine if the findings apply to other mouse cell types and genomic regions.

Taken together, these *in vitro* results in N2a cells indicated that ABE9-SpRY can edit three of the four desired target sites, with increased on-target product purity, two of which could not be generated with ABE8e-SpRY due to bystander mutations. Therefore, we next decided to apply these approaches *in vivo*, testing editing efficiencies in mouse embryos.

### Multiplexed, precise ABE9-SpRY mouse embryo editing

**Fig. 3:**
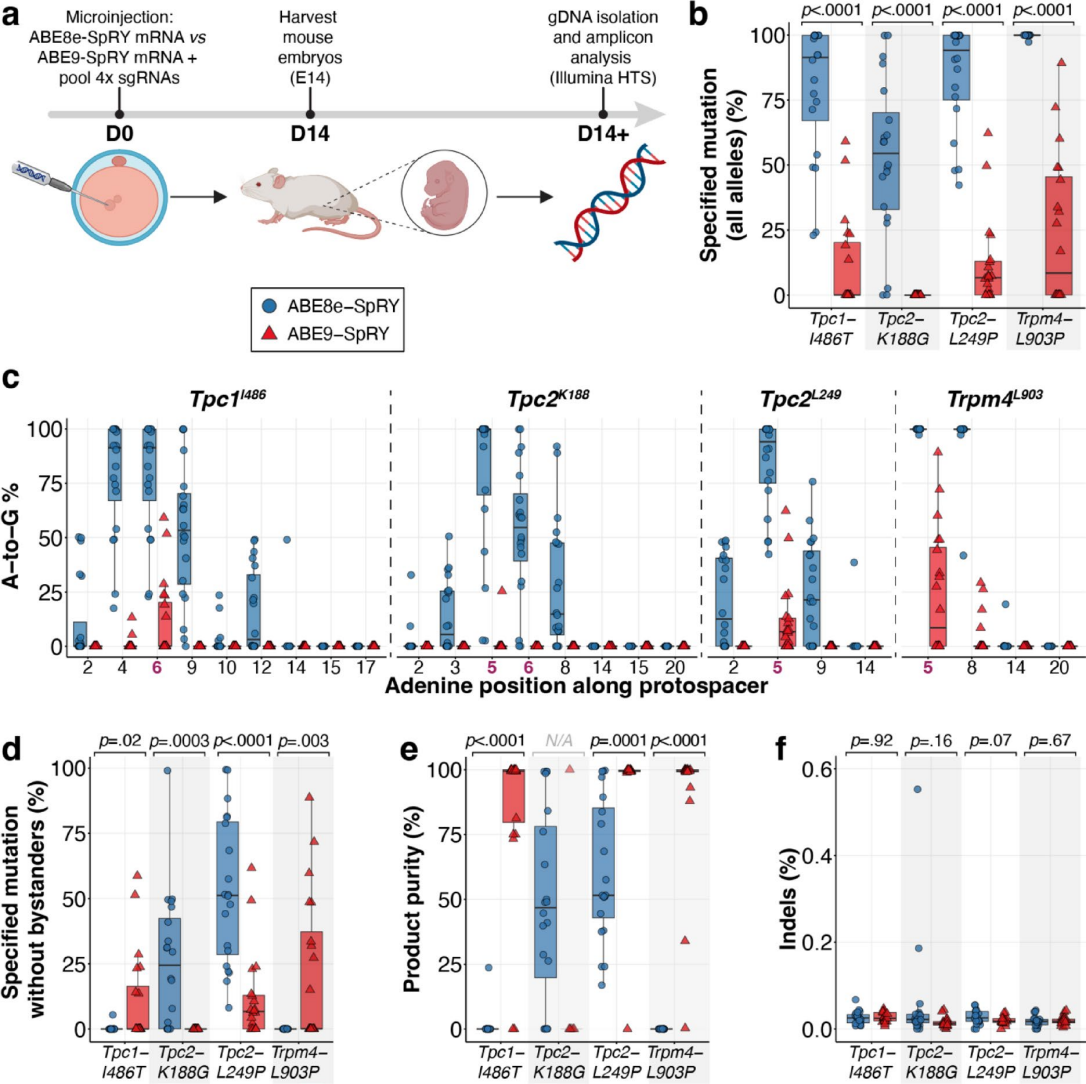
Evaluation of ABE-SpRY constructs at target sites of interest in mouse embryos. (**a**) Schematic of the generation of genetically edited mouse embryos. (**b**) Maximum editing efficiency observed for the target adenine. The “specified mutation (all alleles)” includes all reads that carry the intended substitution, regardless of bystander edits within the analysed window. (**c**) A-to-G editing frequencies in twenty ABE8e or ABE9-SpRY edited mouse embryos at each adenine position along the protospacer region for the four target sites. Target adenines are bolded in magenta. (**d**) Editing efficiency of the target adenine. The “specified mutation without bystanders” reports only clean desired-only (“perfect”) alleles within the analysed window. (**e**) Product purity was calculated as the percentage of reads carrying the desired substitution that were free of bystander edits within the quantification window: 100 × (desired-only / (desired-only + "desired + bystander")). (**f**) Frequency of indel occurrence at the four target sites. Data are displayed as standard boxplots following the Tukey convention, including the median (horizontal line), and individual data points are shown for 20 independent biological replicates. Parts of the schematics in (**a**) were created with BioRender.com.

Adenine base editing in mouse zygotes has been successfully performed using a combination of ABE mRNA and either *in vitro* transcribed or chemically synthesised modified sgRNAs, injected into the cytoplasm or pronucleus of the zygote^40,50–55^. Indeed, these efforts have been highly effective in achieving nearly 100% editing with ABE7.10^50–52^. However, precision issues were apparent, as only 1.3% to 20% of the desired edits occurred within an overall A-to-G efficiency of 95% in the protospacer^51^. To evaluate the *in vivo* editing efficiency of our approach in mouse embryos, we microinjected a pool of four synthetic sgRNAs along with either *in vitro*-transcribed ABE8e-SpRY or ABE9-SpRY mRNA into the cytoplasm of mouse zygotes. Pooling sgRNAs in embryos is an effective screening strategy that reduces animal use and enables locus prioritisation. The injected zygotes were transferred to pseudopregnant surrogate mothers, isolated on embryonic day 14, and their genomic DNA was extracted for targeted deep sequencing analysis (Fig. 3a).

Our analysis indicated that ABE8e-SpRY effectively edited target adenines at all four sites, achieving editing efficiencies of up to 100%. The average editing efficiencies were 78.9 ± 25.7% at *Tpc1-I486* [median (IQR), 91.4% (67.0–99.9%)], 52.6 ± 30.8% at *Tpc2-K188* [median (IQR), 54.5% (32.9–70.1%)], 84.5 ± 20.2% at *Tpc2-L249* [median (IQR), 94.1% (75.1–99.9%)], and 99.8 ± 0.6% at *Trpm4-L903* [median (IQR), 99.9% (41.7–100.0%)], (Fig. 3b). Editing by ABE9-SpRY was observed at *Tpc1-I486T*, *Tpc2-L249*, and *Trpm4-L903P*, achieving efficiencies of up to 59.1%, 62.3%, and 89.3%, with corresponding averages of 11.0 ± 18.1% [median (IQR), 0.04% (0.02–20.26%)], 11.9 ± 16.8% [median (IQR), 6.71% (0.04–12.95%)], and 23.7 ± 28.6% [median (IQR), 8.50% (0.03–48%)], respectively.

A closer examination of adenine base editing across the protospacer sequences revealed a pattern consistent with previous *in vitro* findings. Despite the high editing efficiency of ABE8e-SpRY, its activity was broadly distributed, targeting adenines from positions A2 to A14 across all tested loci (Fig. 3c). In contrast, ABE9-SpRY exhibited a narrower editing window, concentrating edits primarily within positions A4 to A8. Notably, low-level editing was detected in a single embryo at the *Tpc2-K188* locus, where ABE9-SpRY had shown no *in vitro* activity. This editing occurred at only one of the two targeted adenines, resulting in a 25.2% conversion that introduced a p.K188E substitution instead of the intended p.K188G mutation.

Using read-level allele classification within the predefined CRISPResso2 quantification window (Methods), we quantified the fraction of desired-only reads (desired substitution without bystander edits within the window). ABE9-SpRY produced higher desired-only fractions at *Tpc1-I486* (10.7 ± 17.5% vs 0.3 ± 1.2%) and at *Trpm4-L903* (21.3 ± 28.4% vs 0.0 ± 0.0%), whereas ABE8e-SpRY showed higher desired-only fractions activity at *Tpc2-K188* (25.5 ± 25.9% vs 0.0%) and *Tpc2-L249* (52.8 ± 27.5% vs 11.8 ± 16.2%) (Fig. 3d). Analysing product purity under pooled sgRNA injections within the same quantification window revealed significantly higher purity for ABE9-SpRY at the active loci *Tpc1-I486* (85.1 ± 30.6% vs 1.2 ± 5.3%), *Tpc2-L249* (94.6 ± 22.3% vs 60.8 ± 27.1%), and *Trpm4-L903* (90.5 ± 20.8% vs 0.0%) (Fig. 3e). As these measurements were obtained under pooled sgRNA delivery, single-locus delivery may alter editor occupancy and bystander profiles. Indel frequencies were low and comparable between editors (Fig. 3f).

Only one C-to-T conversion was noted at the *Tpc1-I486* locus in an embryo edited with ABE8e-SpRY, occurring at a frequency of 12.6% at position C6, alongside a single instance of 34.7% unwanted A-to-C editing (Supplementary Fig. 2a). Additionally, an unexpected proximal edit outside the protospacer was found at position A-2 of the *Tpc2-L249* locus (Supplementary Fig. 2b). This edit appeared in one ABE9-SpRY and one ABE8e-SpRY injected embryo at frequencies of 12.3% and 21.9%, respectively. A possible explanation is the dynamic nature of the Cas9-sgRNA-DNA complex and conformational changes during editing, which may briefly expose adjacent bases to the deaminase domain, leading to unintended deamination events.

These findings suggest that ABE9-SpRY can enhance editing purity, albeit with locus-dependent activity. Product purity metrics in this study are defined relative to the predefined quantification window and are based on read-level allele classifications within the sequenced amplicon. While this approach captures co-occurrence of desired and bystander edits within the amplicon, it does not provide long-range haplotype phasing beyond the sequenced region; accordingly, additional long-amplicon and phased analyses at selected loci would be valuable to further characterise haplotype-resolved editing outcomes. To further assess precision beyond these target loci, we next analysed Cas9-dependent and Cas9-independent off-target editing to characterise the specificity profiles of these two editors.

### ABE9-SpRY substantially decreases off-target effects compared to ABE8e-SpRY

Cas-dependent off-target events arise from the tolerance of mismatches between sgRNA and the targeting sequence. Although SpRY can target a broader range of genomic sites, its relaxed PAM tolerance also implies that the potential Cas-dependent off-target sites are substantially expanded, as sgRNA-DNA mismatches become the sole determinant of off-target binding without PAM constraints. To assess specificity, we combined ACEofBASEs-predicted^56^ DNA-site panels in mouse embryos with an orthogonal R-loop assay in HEK293T cells, providing complementary first-line assessments at the examined sites. Accordingly, we selected the top five predicted off-target sites for each locus and analysed these across three embryos per group.

**Fig. 4:**
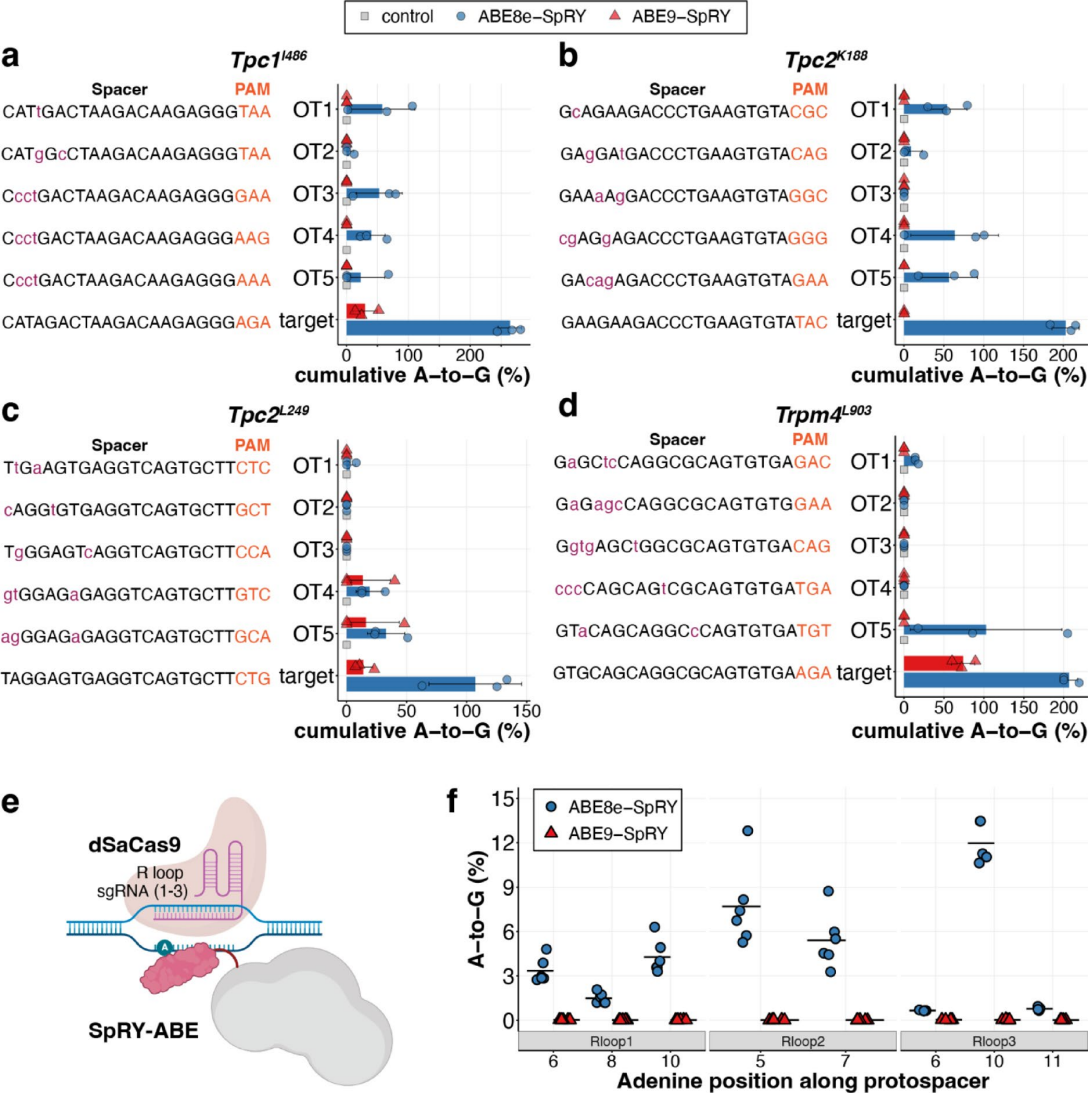
Off-target mutation analysis of ABE-SpRY constructs. (**a**–**d**) Evaluation of cumulative on- and off-target DNA editing by ABE8e- and ABE9-SpRY in mouse embryos in a Cas9-dependent manner. Cumulative editing results from the sum of all A-to-G editing efficiencies within the spacer sequence. Control, gDNA from ear biopsy samples of adult wild-type mice. Individual data points for three independent biological replicates are shown. Error bars indicate standard deviation (SD); bounds were capped at 0% and 100% where the mean ± SD exceeded the valid range. Mismatches in the spacer sequence between on- and off-target sites are displayed in lowercase and magenta. (**e**) Diagram of the R-loop assay illustrating how the base editor can target transient non-target R-loops formed by dSaCas9. (**f**) Cas9-independent assessment of off-target DNA editing by ABE8e-SpRY and ABE9-SpRY, using the orthogonal R-loop assay at specific R-loop sites with five or six biological replicates. The black horizontal bar shows the mean at each adenine position grouped by editor. Parts of the schematics in (**e**) were created with BioRender.com.

When analysing the cumulative percentage of reads containing edits in the off-target spacer sequences, we found that the average cumulative A-to-G editing in the off-target protospacer at the *Tpc1-I486* off-target sites varied from 4.1 ± 5.7% to 57.8 ± 52.8% for ABE8e-SpRY, whereas the corresponding editing levels observed with ABE9-SpRY were comparable to those detected in wild-type control samples (Fig. 4a, Supplementary Fig. 3). Off-target editing at the highest edited adenines ranged from 4.1 ± 7.0% at OT2 to 27.8 ± 17.4% at OT3 for ABE8e-SpRY (Supplementary Fig. 3). Overall, across 20 off-target sites with various PAM sequences, ABE8e-SpRY induced A-to-G edits above control levels at 14 sites, especially at protospacer positions A2 to A20, with editing efficiencies of up to 60.8%, 32.1% and 84.1% for OT4 of *Tpc2-K188*, OT4 of *Tpc2-L249* and OT5 of *Trpm4-L903*, respectively (Fig. 4a–d, Supplementary Fig. 3–6, Supplementary Table 11). However, the variation between the replicates was high, leading to average cumulative A-to-G editing efficiencies from 2.7 ± 4.6% (*Tpc2-L249*, OT1) to 102.7 ± 94.8% (*Trpm4-L903*, OT5). In contrast, ABE9-SpRY edited just two off-target sites associated with the *Tpc2-L249* locus, reaching 40.0% and 48.1% editing, respectively (Fig. 4c, Supplementary Fig. 5). Strikingly, for these two off-target sites, the corresponding embryos’ on-target editing efficiencies at the locus were relatively low, at 7.4% and 10.7%. While off-target effects are known to occur with base editors, this drastic skew towards off-target editing (with a 3-base mismatch) is remarkable. Since these events were observed in just one replicate of ABE9-SpRY, the data indicate that ABE9-SpRY offers improved precision outcomes at these loci, though rare, sequence-dependent off-target activity may still occur.

In addition to Cas-dependent off-target effects, base editors have also been reported to exhibit Cas-independent off-target activity, which is evoked by the deaminase activity of the editors^21,22^. We therefore asked whether TadA-8e and TadA-9 deaminases cause Cas-independent off-target effects to similar extents, regardless of the Cas9 nickase variant to which they are attached. To this end, we employed the orthogonal R-loop assay^57^ (Fig. 4e), which creates a stable R-loop structure at a specific genomic site, independent of the target region, by using an orthogonal DNA-binding module. This exposes a short ssDNA segment without recruiting the tested base editor via sgRNA/Cas9. The base editor is then expressed *in trans*, and any A-to-G conversion within the exposed ssDNA reports Cas-independent deamination.

In HEK293T cells, FACS-enrichment of GFP co-transfected cells showed no editing across eight adenines and three R-loop spacers with ABE9-SpRY, similar to the results reported earlier for ABE9^24^. In contrast, ABE8e-SpRY caused substantial editing from 4.3 ± 1.1% (R loop 1), 7.7 ± 2.7% (R loop 2), to 12.0 ± 1.4% (R loop 3) (Fig. 4f). These results highlight the high specificity of ABE9-SpRY despite the increased non-specific binding of SpRY.

### ABE9-SpRY generated precise founders at two loci with high editing and transmission at the targeted sites

**Fig. 5:**
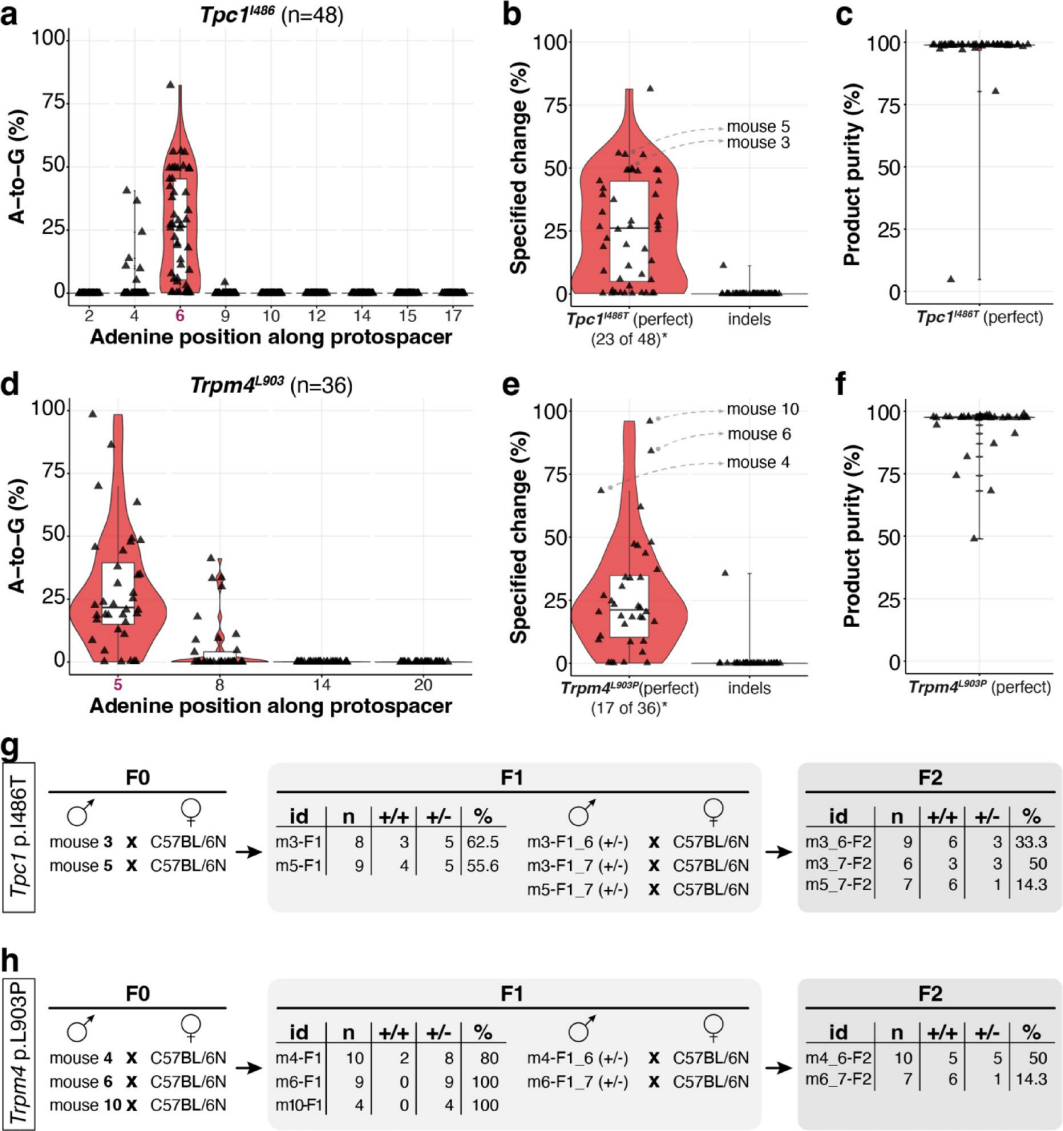
ABE9-SpRY can effectively produce *Tpc1*^*I486T*^ and *Trpm4*^*L903P*^ founder mice. A-to-G editing frequencies plotted across each adenine position are shown in (**a**) for 48 *Tpc1*^*I486*^ adult mouse biopsies, and (**d**) for 36 *Trpm4*^*L903*^ adult mouse biopsies. The frequency of desired editing (without bystander edits) and indels are presented in (**b**) for *Tpc1*^*I486*^ adults, and (**e**) for *Trpm4*^*L903*^ adult mice. Product purity is calculated as the percentage of correctly edited sequences relative to the total number of modified alleles and is displayed in (**c**) for *Tpc1*^*I486*^ adults, and (**f**) for *Trpm4*^*L903*^ adult mice. Data are presented as standard boxplots (white) following the Tukey convention, which includes the median (black horizontal line), displayed within violin plots (red), and individual data points (black) representing each biological replicate. Crossing schemes of F0 founder mice, with numerical data for F1 and F2 transmission for *Tpc1*^*I486T*^ (**g**), and *Trpm4*^*L903P*^ (**h**), respectively. *Asterisk note* (**b**, **e**): The value shown beneath “perfect” indicates the number of F0 founders classified as high-confidence clean allele-carrying at the target locus, using read-level allele classes within the CRISPResso2 quantification window. High-confidence founders were defined as those with ≥20% desired-only (“perfect”) reads and <2.5% unwanted outcomes(unwanted = "desired + bystander" + bystander-only + indels). Full per-founder allele-class compositions and the classification are provided in Supplementary Fig. S9.

Accurate base conversion is crucial for modelling SNVs; therefore, minimising bystander and off-target mutations is essential. Given the negligible levels of bystander editing, low Cas9-dependent off-target editing, and no evidence above background of Cas9-independent off-target editing produced by ABE9-SpRY, we decided to use this editor for generating two individual founder mouse lines harbouring the *Tpc1*^*I486T*^ and *Trpm4*^*L903P*^ mutations. To achieve this, ABE9-SpRY and the locus-specific sgRNAs were injected separately into mouse zygotes. Analysis of adult mice biopsies from 48 putative F0 founder mice revealed robust editing efficiencies (Fig. 5a,b), with the precise p.I486T edit with fewer bystander edits and indels achieved at 25.6 ± 21.0% efficiency [median (IQR), 26.1% (4.9–44.7%)], and up to 81.4% (Fig. 5b). The very low rate of bystander editing at A4 (Fig. 5a) resulted in a considerable product purity of 96.4 ± 13.8% [median (IQR), 98.9% (98.8–99.0%)] (Fig. 5c). While one individual mouse exhibited an indel frequency of 11.2% at the *Tpc1-I486* locus, overall indel levels were generally minimal (0.3 ± 1.6%) (Fig. 5b). Analysis of 36 *Trpm4-L903* ABE9-SpRY edited mice showed a similar trend (Fig. 5d,e). The product purity was comparable, as observed for *Tpc1-I486*, at 93.9 ± 10.3% [median (IQR), 97.6% (97.4–97.8%)] (Fig. 5f), due to the limited bystander editing events at A8 (Fig. 5d). In ear biopsies, p.L903P editing fractions exceeded those observed for p.I486T, at frequencies of 28.3 ± 20.8% [median (IQR), 21.2% (10.4–34.7%)], and up to 96.0% (Fig. 5b,e). However, the degree of somatic mosaicism in F0 founders may influence the relationship between ear-biopsy allele fractions and germline composition. Similarly, indel frequencies were slightly higher at the *Trpm4-L903* locus at 1.0 ± 5.9%, reaching 35.5% in one instance (Fig. 5e). Using a predefined operational threshold (desired-only ≥ 20% and unwanted outcomes ≤ 2.5% within the quantification window; unwanted = "desired + bystander" + bystander-only + indels), we further reported the number of high-confidence clean allele-carrying founders for each locus of 23 and 17, for *Tpc1* p.I486T and *Trpm4* p.L903P, respectively (Fig. 5b,e; Supplementary Fig. S9).

To make read-level co-occurrence of desired and bystander edits explicit, we classified amplicon reads within the CRISPResso2 quantification window into WT, desired-only (“perfect”), desired + bystander(s), bystander-only, and indel-containing categories (Supplementary Fig. S9), with representative allele frequency spectra (Supplementary Fig. S10).

To determine the F1 transmission rate and establish stable mutant lines for *Tpc1* p.I486T and *Trpm4* p.L903P, we proceeded to mate two verified adult F0 *Tpc1* p.I486T male mice (Fig. 5g) and three verified adult F0 *Trpm4* p.L903P male mice (Fig. 5h) with wild-type females. We observed high transmission rates for *Tpc1* p.I486T of 55.6% and 62.5% in the two independent matings (Fig. 5g, Supplementary Fig. 7a), and high to complete transmission for *Trpm4* p.L903P at rates ranging from 80 to 100% (Fig. 5h, Supplementary Fig. 7b). Finally, we mated selected heterozygous F1 carriers with wild-type mice to establish stable lines (Supplementary Fig. 8).

These results suggest that the pooled embryo analysis has somewhat underestimated the potential editing efficiency of ABE9-SpRY when deployed to individual loci rather than diluted across several for screening. The high editing efficiencies achieved, up to 96% at one target site, underscore the utility of ABE9-SpRY for establishing stable mouse lines at the loci tested. While somatic mosaicism in the F0 generation necessitates the use of F1 offspring for standardised phenotypic characterisation, the observed F0-F1 transmission supports germline contribution, although ear-biopsy allele fractions may not fully reflect germline composition or uniquely determine the degree of mosaicism. Nonetheless, the high frequency of transmissible desired alleles streamlines the transition from zygote injection to the establishment of the experimental cohort.

### Precise hiPSC engineering of *TPC1* with ABE9-SpRY

**Fig. 6:**
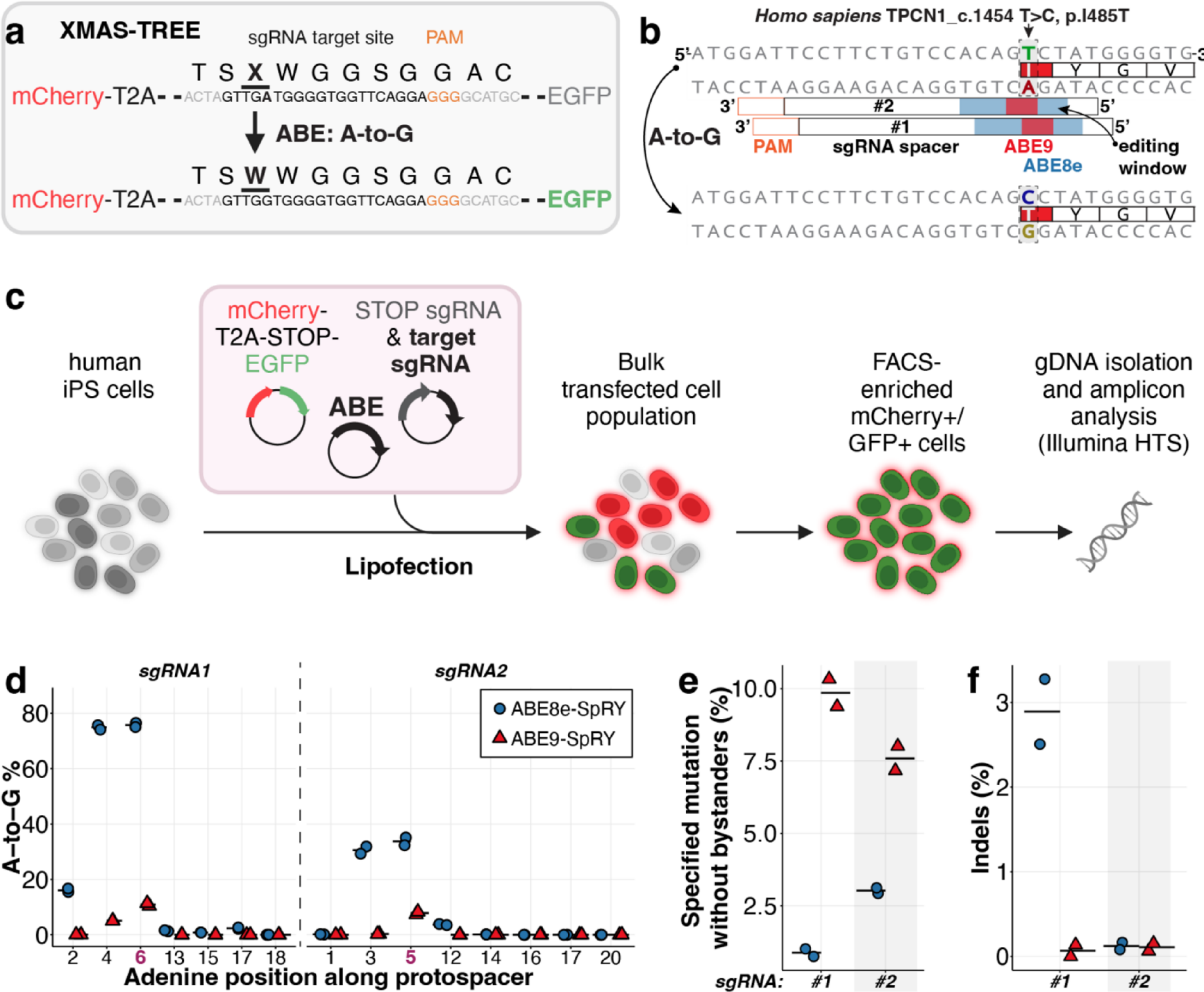
Enrichment and analysis of base-edited hiPSC populations via XMAS-TREE. (**a**) Diagram of the XMASTREE system, illustrating the expression of an mCherry cassette followed by a stop codon (TGA) and a GFP cassette. Conversion of the adenine in the stop codon to guanine activates GFP protein expression. (**b**) Diagram showing the tiling of two sgRNAs across the target genomic region. (**c**) Schematic of the XMASTREE workflow for identifying and enriching adenine base-edited cells. Flow cytometry was used to isolate populations that are double-positive for mCherry and GFP signal for downstream sequencing. (**d**) Percentage of adenine-to-guanine conversions along the protospacer. (**e**) Editing efficiency of the target adenine. The “specified mutation without bystanders” reports only clean desired-only (“perfect”) alleles within the analysed window. (**f**) Percentage of indels observed with ABE8e-SpRY or ABE9-SpRY, using sgRNA1 or sgRNA2 in sorted cells. Horizontal bars represent the mean from two biological replicates. Parts of the schematics in **c** were created with BioRender.com.

Over the past two decades, hiPSCs have become a powerful *in vitro* model, offering a human-specific system for studying disease mechanisms, drug responses, and genetic variants in a physiologically relevant context when paired with directed-differentiation procedures^58,59^. Given this importance, we aimed to validate the potential of ABE9-SpRY for disease modelling in hiPSCs. To enrich base-edited cells, we used the XMAS-TREE assay^60^, in which a successful A-to-G conversion of a stop codon in between an episomal mCherry-linker-EGFP construct leads to the co-expression of GFP alongside the pre-existing mCherry signal (Fig. 6a). We selected the human p.I485T mutation of human *TPC1*, corresponding to mouse *Tpc1* p.I486T, as a test and designed two sgRNAs with the target adenine located at position A5 (sgRNA#2) or A6 (sgRNA#1) of the protospacer sequence, respectively (Fig. 6b). We then co-transfected either ABE8e-SpRY or ABE9-SpRY plasmid, which we re-cloned under an EF1α promoter for robust hiPSC expression, along with the reporter plasmid, a reporter-STOP-sgRNA plasmid, and either of the two target sgRNA plasmids, thereby enriching the base-edited cell population through FACS sorting (Fig. 6c). FACS analysis showed that within the mCherry⁺ population, 59.3–64.3% of cells were GFP⁺ using ABE8e-SpRY, while 50.3–53.1% were GFP⁺ using ABE9-SpRY, depending on the sgRNA used (Supplementary Table S12). This indicates similar levels of reporter activation through A-to-G conversion of the stop codon in the XMAS-TREE reporter in cells transfected with either editor. While the overall editing efficiency was substantially higher with ABE8e-SpRY, 75.8 ± 1.0% editing with sgRNA#1 and 33.7 ± 2.0% with sgRNA#2, ABE8e-SpRY was also active very broadly. In particular, the two adjacent bases A4 and A6, or A3 and A5 for sgRNA#1 and sgRNA#2, respectively, displayed very similar activity. By contrast, ABE9-SpRY achieved efficiencies of only up to 10.9 ± 0.7% with moderate bystander editing at A4 (5.1 ± 0.1%) (Fig. 6d). However, we observed superior overall desired editing activity with minimal unwanted mutations, which could be a result of both higher precision and overall low editing activity of ABE9-SpRY, with sgRNA#1 reaching 9.9 ± 0.7% at the target adenine A6 (Fig. 6e). Notably, despite ideal target site positioning, sgRNA#2 showed a slightly lower efficiency at 7.6 ± 0.6%. The highest editing rate without unwanted mutations for ABE8e-SpRY was 3.0 ± 0.1%. While the overall editing rates of ABE9-SpRY were lower, this limitation is less critical in the context of hiPSC-based disease modelling, where correctly edited clones are typically isolated and expanded prior to downstream differentiation into specific cell types of interest. Importantly, such clonal isolation strategies rely on the high precision of the editing event, which we achieved with ABE9-SpRY, but not ABE8e-SpRY. In addition to the increased frequency of indels observed (Fig. 6f) at 2.9 ± 0.5% with ABE8e-SpRY compared to 0.1 ± 0.1% with ABE9-SpRY using sgRNA#1, these results provide preliminary evidence for the potential applicability of ABE9-SpRY in precision disease modelling applications, spanning from animal to human cellular models.

## Discussion

Base editing holds great promise for modelling point mutations due to its benefits over existing techniques, as it prevents DSBs and shows high on-target efficiency^15,16^. While previous studies have utilised base editing for animal modelling, they have frequently encountered issues such as off-target effects, bystander editing, and indels^50,51,61,62^.

For interpretable disease modelling, base editing should minimise (1) bystander missense mutations, as they can easily mask or disrupt the interpretation of the desired mutation^63^, (2) indels on the edited allele to avoid frameshifts and truncated proteins^64^, and (3) off-target edits, which can persist despite outcrossing^65^ and may require genome-wide validation.

Here, we leveraged recent advances in base editing by fusing ABE9, an editor with a narrower activity window and improved product purity^24^, to the PAM-flexible SpRY-Cas9 nickase^25^. Across the loci tested, ABE9-SpRY introduced A-to-G substitutions with broad targetability and a high fraction of desired-only alleles within the predefined quantification window, while exhibiting low indel levels and fewer Cas-dependent off-target events at the predicted sites examined. In mouse embryos, desired-only editing reached up to 89% at individual loci. Moreover, in adult F0 founder mice, we observed up to 96% desired editing at the *Trpm4-L903* locus, highlighting the potential for phenotyping within the same generation. In contrast, ABE8e-SpRY exhibited a higher overall A-to-G conversion efficiency, close to homozygosity. However, this was accompanied by bystander mutations at similar frequencies, resulting in significantly lower product purities. Furthermore, ABE8e-SpRY caused substantial levels of Cas-dependent off-target editing in mouse embryos at 14 of 20 sites examined, with A-to-G editing frequencies at OT5 of the *Trpm4-L903* sgRNA reaching up to 84%. Therefore, the trade-off between reduced editing efficiency and high precision remains a valuable advantage that ABE9-SpRY offers for generating genetic and disease models where bystander editing is unacceptable.

We found that pooling sgRNAs during the initial *in vivo* testing in embryos is an effective strategy. This approach enables rapid screening of editing outcomes with reduced effort and decreases the number of animals required. However, this approach seems to underestimate editing efficiency compared to experiments where only a single sgRNA is injected. For example, at the *Tpc1-I486* locus, perfect editing (desired editing without bystander edits) occurred at a frequency of 10.7 ± 17.5% in embryos (pooled) versus 25.6 ± 21.0% in adults (single). Similarly, at the *Trpm4-L903* locus, the desired editing frequency was 21.3 ± 28.4% (pooled) versus 28.3 ± 20.8% (single). This discrepancy may stem from the dilution of the editing machinery across four different target sites, and potentially their off-targets, in the pooled condition. This likely reduces the effective concentration of the editor at each individual locus, thereby lowering the observed editing efficiency. Furthermore, competitive interference among the pooled sgRNAs for the limited concentration of the base editor protein, combined with locus-specific factors such as chromatin accessibility and sgRNA secondary structure, likely contributes to this reduced performance. In practice, pooled designs may be improved by adjusting sgRNA ratios based on prior single-guide *in vitro* performance (e.g., increasing the proportion of weaker guides), thereby improving the likelihood of recovering informative edits while minimising animal use. Consequently, while pooling serves as an effective initial screening tool, single-locus validation is essential to determine the maximum potential editing efficiency of a given guide. More broadly, editing efficiency and purity are sequence- and locus-dependent, and generality across additional genomic sites remains to be established.

While our founder mice showed high editing and transmission, the exact degree of somatic mosaicism in each F0 remains unknown. Given the unknown and locus-dependent mosaicism in these founders, and the evidence that *Tpc1* p.I486T founders transmit edits in a pattern more consistent with functional heterozygosity, whereas *Trpm4* p.L903P founders more closely resemble homozygosity, phenotyping F0 animals as if they were stable heterozygotes or homozygotes is not justified. Instead, established F1 lines should be utilised for definitive phenotypic characterisation to ensure genotype stability across tissues.

At the *Tpc2-K188* locus, ABE8e-SpRY demonstrated efficient editing, while only one mouse embryo showed any editing for ABE9-SpRY. This indicates that the SpRY-Cas9n protein can access this target site, but the evolved TadA-9 deaminase enzyme has shown reduced substrate acceptance compared to TadA-8e. Given that the N108Q and L145T mutations in ABE9 are speculated to expel the backbone of the DNA substrate^24^, this may also change the substrate-interaction behaviour of TadA-9 compared to TadA-8e, in addition to altering the editing window. As such, the observation at the *Tpc2-K188* locus suggests that, in addition to altering the editing window, these mutations may create sequence-dependent steric hindrances that prevent the enzyme from effectively engaging with the target adenine, even within its supposedly optimal editing window. To better understand the genome-wide targeting properties of ABE9-SpRY, a combination of detailed structural analyses to elucidate the mechanisms by which the N108Q and L145T mutations influence substrate binding and high-throughput *in vitro* analysis of a large number of disparate target sites will be required. However, despite its efficiency, ABE8e-SpRY would not be the alternative to ABE9-SpRY in cases such as those at the *Tpc2-K188* locus due to its off-target profile. While a recent benchmarking study has included ABE9-SpRY in broader editor comparisons^66^, our study focuses on practical deployment and outcome profiling of ABE9-SpRY at disease-relevant loci for bystander-minimised mouse line generation and a single-locus hiPSC proof-of-concept. Where ABE9-SpRY activity is limiting, alternative higher-specificity ABE variants may be preferable. These include earlier reported engineered ABE8e variants (e.g. ABE8e-WA/WQ^33^, ABE8e-N108Q^24^, ABE8e-V106W^23^) and more recently reported ABEs (e.g., ABE8r^67^, ABE-Umax^68^, V28C/ABEx1^66^, E2-/E4-ABE^69^, TadA-8e-L1B_S116^70^, hpABE5.20^71^), which may serve as suitable substitutes when fused to SpRY-nickase and should be evaluated in embryos and *in vivo*, including their off-target profiles. In practice, scalable machine learning-based engineering approaches to create bespoke ABE-SpCas9 variants with target-specific PAM preferences^72^ may further improve on-target activity while reducing off-target editing.

The PAM-less properties of SpRY, when used with next-generation genome editors, pose notable challenges, particularly increased off-target genome editing, which can result in more chromosomal rearrangements^73^. As a result, ABE-SpRY editors are expected to exhibit increased off-target editing because they can bind and modify DNA sequences without the stringent requirement for a PAM. ABE8e-SpRY demonstrated extensive Cas9-dependent off-target editing at 70% of the examined off-target sites. At one of these sites, *Trpm4-L903* OT5, editing reached up to 84%. ABE9-SpRY, in contrast, exhibited editing at only 10% of the examined sites. We acknowledge that a limitation of our off-target analysis is its dependence on *in silico* predictions of potential non-specific sgRNA binding sites. This method may introduce bias, as the sites are selected a priori and might miss *bona fide* off-target effects. Nonetheless, it remains a common and practical initial method for evaluating base editor specificity. To complement *in silico* predictions, a suite of unbiased genome-wide off-target detection methods has emerged in recent years^74^. For adenine base editors, specific unbiased genome assays like EndoV-seq^75^, Digenome-seq^76^, and Selicit-seq^77^ have been developed. Especially when stringent validation is necessary, such as in clinical settings, these should be complemented with detailed chromosome-level structural analyses using assays like CAST-seq^78^. Although these advanced methods are valuable, our results using *in silico* prediction already demonstrate that ABE9-SpRY strikes an attractive balance of precision and flexibility, supporting its suitability for generating disease models.

It is important to note that, across our tested loci, higher product purity with ABE9-SpRY often coincided with lower overall A-to-G conversion compared with ABE8e-SpRY. Reduced catalytic activity can decrease opportunities for bystander edits; therefore, the observed precision likely reflects a combination of the narrowed deamination window and overall activity. We did not perform dose/time titrations to match on-target editing frequencies between editors; activity-matched comparisons will be useful for further disentangling these contributions. Moreover, as F0 founders can be mosaic, genotypes inferred from ear-biopsy amplicon sequencing may not fully reflect germline composition.

While our off-target analyses provide a strong first-line assessment of specificity, we did not perform unbiased genome-wide DNA or RNA off-target mapping or structural-variant detection in embryos, founders, or hiPSC clones; in particular, genome-wide SNV/SV profiling (e.g., WGS) was not performed on founder animals. Given SpRY’s PAM-flexible nature, future work, including genome-wide DNA/RNA off-target profiling and SV detection, will be important for defining the genome-wide off-target risk profile.

hiPSCs are widely used for disease modelling, but such applications require highly precise variant installation^79,80^. As a single-locus proof-of-concept, we targeted the endogenous *TPC1* p.I485T site with two tiled sgRNAs and observed fewer bystander and indel events with ABE9-SpRY than ABE8e-SpRY under our tested conditions. Because editor dose and expression were not titrated to achieve matched on-target editing, these differences may partly reflect the lower overall activity of ABE9-SpRY. Future work should evaluate additional loci and include activity-matched comparisons.

Overall, ABE9-SpRY demonstrates high precision while maintaining considerable flexibility, which is crucial, as missense mutations can be highly detrimental. For example, in the context of disease rescue, even with ABE8e’s high efficiency in introducing the intended point mutation, a bystander edit caused a missense mutation that ultimately prevented phenotypic rescue^63^. This underscores the importance of precision editors like ABE9-SpRY, particularly when modelling diseases or designing therapeutic strategies where functional outcomes are paramount. This level of precision is essential for fundamental research, such as structure–function studies of a specific protein domain, where even a single unintended amino acid change introduced by bystander editing could make the results uninterpretable. The potential for base editors to introduce confounding SNVs, as systematically demonstrated in mouse embryos^62^, underscores the need for cleaner tools like ABE9-SpRY to produce reliable models for functional analysis. With ABE9-SpRY’s versatility in targeting specific adenines at amenable loci, along with minimised bystander editing and reduced Cas-dependent and Cas-independent off-target effects, it represents a valuable tool for creating precise disease models in both animals and cell lines, hiPSCs, and organoids derived from them.

## Methods

All methods were performed in accordance with the relevant guidelines and regulations.

### Reagents and biological resources

Information on major resources is provided in Supplementary Table 1.

### Mouse strains

Wild-type C57BL/6N mice were purchased from Charles River Laboratories (Wilmington, MA, USA). The experimental procedures were approved by the regional council of Karlsruhe, Germany, in accordance with the Animal Welfare Act (AZ35-9185.81/G-60/23). Microinjection was performed in the cytosol of C57BL/6N zygotes. All animals were housed in the Interfaculty Biomedical Faculty (IBF) of Heidelberg University. Mice were maintained under specified pathogen-free conditions with a 12-h light/12-h dark cycle, and water and standard food (Rod18, LASvendi GmbH, Germany) available ad libitum. All mouse experiments described in this study comply with ARRIVE guidelines.

### Identification and alignment of mouse orthologues for targeted point mutations

To identify the corresponding mutations in the mouse genome, to four target loci of interest with missense mutations *Tpc1 p.I485T*, *Tpc2 p.L265P*, *Tpc2 p.K204A*, and *Trpm4 p.L907P* we retrieved the *Mus musculus* and *Homo sapiens* gene sequences from Ensembl and performed protein sequence alignments. This allowed us to pinpoint the target amino acids and the corresponding adenines in the mouse coding sequence. Through this alignment, we identified the orthologous amino acid residues in the respective mouse genes and the potential missense mutations we can model by A-to-G transition mutation mediated through ABEs: *Tpc1 p.I486T*, *Tpc2 p.L249P*, *Tpc2 p.K188G*, and *Trpm4 p.L903P*. Notably, for *Tpc2 p.K188G*, a glycine substitution was created *in silico* instead of the alanine. We decided to proceed with the glycine substitution based on the similar chemical and functional properties of glycine and alanine.

### Design and cloning of sgRNAs

sgRNAs were designed manually to enable the precise installation of the defined missense mutations and validated using ACEofBASEs^56^ with the following parameters: select BaseEditor “custom ABE” with Window start "3" and Window end “11” with PAM “NRN” for *Tpc1-I486*, *Tpc2-K188*, and *Trpm4-L903* target sites or with PAM “NYN” for the *Tpc2-L249* target site; for Species “Mouse (Mus musculus) Ensembl V 103”. The full list of sgRNA oligonucleotides to clone and the sgRNAs used in this study are shown in Supplementary Tables 2 and 3.

For *in vitro* applications of sgRNAs, the 20 bp SpCas9 sgRNA spacer sequences used in this study were designed with the 5’ and 3’ sequences (Supplementary Table 2), as previously described^81^, with an additional 5’ “G” added in cases in which the spacer does not start with a G. In brief, the 20 bp target site (N20) sequences were ordered as oligonucleotides with the following sequences 5’-CACC(N20-21)GTTTT-3’, while the sequences complementary to the target site were designed and ordered as oligonucleotides as follows 5’-CTCTAAAAC(N20-21)-3’. sgRNAs were cloned into the BsaI-digested 2.18 kb pU6-pegRNA-GG-acceptor fragment (pU6-pegRNA-GG was a gift from David Liu, Addgene plasmid #132777^81^) by oligonucleotide annealing and Golden Gate assembly as previously described^81^, including the standard SpCas9 sgRNA scaffold.

*R-loop SaCas9 sgRNAs.* To generate the SaCas9 sgRNA plasmid, BPK1520, containing a pU6_BsmBIcassette-Sp-sgRNA was used as a backbone (BPK1520 was a gift from Keith Joung, Addgene plasmid #65777^42^). The SpCas9 sgRNA scaffold was digested using *BsmBI* and *HindIII*, and the digested fragment was isolated. The SaCas9 sgRNA scaffold sequence^45^, designed with complementary overhangs (Supplementary Table 2), was ligated into the digested vector backbone. The designed sgRNA spacer sequences were then ordered as oligonucleotides with *BsmBI* overhangs (Supplementary Table 2), and the annealed oligos were cloned into the *BsmBI*-digested SaCas9 sgRNA plasmid via Golden Gate assembly. In brief, in a 10 µl reaction volume, 50 ng of SaCas9 sgRNA plasmid, 10 units of *BsmBI*-v2 (NEB), 20 units of T4 DNA ligase (NEB), 1 µL of 1 µM annealed spacer oligonucleotides, 1X T4 DNA ligase buffer were assembled under the following thermal cycling conditions: digestion at 42 °C for 4 min, followed by ligation at 16 °C for 3 min, repeated for a total of 11 cycles. The reaction was then incubated at 80 °C for 10 min to inactivate *BsmBI*.

*hiPSC sgRNAs.* Cloning of targeting sgRNA into the pDT-sgRNA-XMAS-1 × plasmid was performed as previously described (pDT-sgRNA-XMAS-1 × was a gift from Xiao Wang, Addgene plasmid #164413^60^).

All sgRNAs for mouse experiments were ordered via Integrated DNA Technologies (IDT) as custom Alt-R CRISPR-Cas9 sgRNA (Supplementary Table 3).

### Cloning of adenine base editor plasmids

All plasmids constructed here were cloned via Gibson Assembly using the NEBuilder HiFi DNA Assembly (NEB) kit with PCR products amplified from designated template plasmids with specific primers (Supplementary Table 4) with Q5 Hot Start DNA polymerase (NEB).

To obtain pCMV_ABE9, we used the ABE9 editor sequence form^24^, and recloned pCMV_ABE8e (a gift from David Liu, Addgene plasmid #138489^23^) by introducing the two defining mutations N108Q and L145T by PCR, altogether assembled via four fragments (Supplementary Table 4): pCMV_backbone, ABE9_fragment_1_TadA*_Nterm_N108Q, ABE9_fragment_2_TadA*_N108Q_L145T, ABE9_fragment_3_Cas9n-Nterm. To clone pCMV_ABE8e-SpRY, we assembled the following PCR products: pCMV_backbone, ABE8e_fragment1_TadA*, and ABE9-SpRY_fragment_4_SpRY-Cas9n (amplified from pCMV-T7-ABE8e-nSpRY-P2A-EGFP (KAC1069), a gift from Benjamin Kleinstiver, Addgene plasmid #185912^82^). Similarly, pCMV_ABE9-SpRY was constructed by assembling: pCMV_backbone, ABE9_fragment_1_TadA*_Nterm_N108Q, ABE9_fragment_2_TadA*_N108Q_L145T, ABE9_fragment_3_Cas9n-Nterm, and ABE9-SpRY_fragment_4_SpRY-Cas9n.

For the transfection of hiPSCs, pCMV_ABE8e-SpRY and pCMV_ABE9-SpRY were recloned to be expressed under the control of the EF1α promoter. In brief, two fragments were amplified, first the pEF1α_backbone (from pEF1α-hMLH1dn, a gift from David Liu, Addgene Plasmid #174824^83^) and then either ABE8e-SpRY or ABE9-SpRY, for subsequent Gibson assembly cloning.

The entire sequence of all constructs assembled using PCR was verified with the PlasmidEZ (Azenta/GeneWiz) full-plasmid sequencing service. All plasmids used for the transfection experiments were prepared using the EndoFree Plasmid Maxi Kit (Qiagen).

### Generation of adenine base editor mRNA

mRNA for the microinjection of mouse zygotes for ABE8e-SpRY and ABE9-SpRY was generated by *in vitro* transcription from *SalI*-HF linearised pCMV_ABE8e-SpRY and pCMV_ABE9-SpRY plasmid templates, respectively, using the HiScribe T7 ARCA mRNA Kit. The mRNA was purified using the RNeasy Mini Kit. mRNA quality was confirmed using the TapeStation RNA ScreenTape.

### Mammalian cell culture and transfection

Human HEK 293 T cells and Neuro2a cells were purchased from ATCC and maintained in Dulbecco’s modified Eagle’s medium (DMEM; Gibco) supplemented with 10% FBS (Gibco) at 37 °C with 5% CO_2_, up to 20 passages, by passaging cells every two to three days. The hiPSC line SCVI15 (Supplementary Table 1) was obtained from Greenstone Biosciences, Inc. via Timon Seeger; beyond the information listed in Supplementary Table 1, no additional donor background or genomic characterisation information was provided by the source. The hiPSCs were cultured on hESC-Qualified Matrix Matrigel-coated (Corning) 6-well plates (Sarstedt) in StemMACS PSC-Brew XF medium (Miltenyi Biotec). Medium was changed daily, and cells were passaged every three days using StemMACS Passaging Solution XF (Miltenyi Biotec). The culture medium was supplemented with 2.5 µM Rock Inhibitor (Y27632, Miltenyi Biotec) on passaging days for 24 h. The supernatant media from cell cultures were routinely analysed for mycoplasma presence using the MycoSPY Master Mix (Biontex).

All HEK293T and N2a transfection experiments were conducted with at least three independent biological replicates. HEK293T and N2a cells were seeded at a density of roughly 110,000 and 180,000 cells per well in 24-well plates, respectively, 20–24 h before transfections. Transfections were performed with 750 ng of base editor plasmid, 250 ng of sgRNA expression plasmid, and, for experiments involving GFP co-transfection, 200 ng of pMAX-GFP (Lonza), combined with 2 μl of Lipofectamine 2000 (Thermo Fisher Scientific) in a total volume of 50 μl Opti-MEM (Thermo Fisher Scientific), according to the manufacturer’s instructions. Medium was exchanged to culture medium approximately 24 h after transfection. For the Orthogonal R-loop assay, HEK293T cells were seeded at a density of 110,000 cells per well in 24-well plates, as described above. However, the cells were co-transfected with a mixture containing 500 ng of plasmids encoding base editor plasmids, 350 ng of SpCas9 sgRNA (*HEKsite2*), 500 ng of dSaCas9, 350 ng of *sa*Cas9 sgRNAs targetting separate genomic loci unrelated to the on-target site, and 200 ng of pMAX-GFP (Lonza).

hiPSCs were seeded on 6-well plates and transfected after approximately 24 h at around 60% confluency. Before transfections, the medium was changed to StemMACS PSC-Brew XF supplemented with StemMACS PSC-Support XF (Miltenyi Biotec). Transfections were performed with 300 ng pEF1α-XMAS-1xStop (a gift from Xiao Wang, Addgene plasmid #164411^60^), 300 ng pU6-SpCas9-XMAS-1 × plasmid, 2100 ng of pEF1α_ABE8e-SpRY or pEF1α_ABE9-SpRY, and 10 µl Lipofectamine Stem (Thermo Fisher Scientific) per well, according to the manufacturer’s instructions. Medium was exchanged to culture medium approximately 24 h after transfection.

### Fluorescence-activated cell sorting

Approximately 48 h after transfections, HEK293T, N2a or hiPSCs were harvested for sorting. HEK293T and N2a cells were treated with TrypLE Express Enzyme (Thermo Fisher Scientific) to singularise the cells, followed by a washing step with D-PBS. The cells were resuspended in buffer containing 1 × PBS without Mg^2+^/Ca^2+^ (Thermo Fisher Scientific), 0.5% FCS (Thermo Fisher Scientific) and 1% Penicillin–Streptomycin and filtered through a 35 µm cell strainer (Corning). hiPSCs were dissociated with Accutase (Thermo Fisher Scientific) and washed with D-PBS, followed by resuspending the cells in 1 × PBS without Mg^2+^/Ca^2+^, 0.5% FCS, 2.5 µM Rock Inhibitor and passed through a 35 µm cell strainer (Corning). HEK293T and N2a cells co-transfected with pMAX-GFP were sorted for the top 30% GFP-positive population. For the XMAS-TREE reporter assay in hiPSCs, only cells co-expressing GFP and mCherry were sorted, and the data were analysed using a sequential gating strategy. Events were first gated as “Cells” based on forward- and side-scatter properties to exclude debris, followed by selection of “Singlets” using FSC-A versus FSC-H to exclude doublets. Within the singlet population, mCherry⁺ cells were identified using thresholds defined by negative and single-colour controls, and the mCherry⁺GFP⁺ double-positive population was subsequently quantified. Representative gating plots and population statistics (%Parent and %Total as reported by the acquisition software) for all samples are shown in Supplementary Fig. 11–13 and Supplementary Table 12. FACS sorting was performed with either a BD FACSAriaIII or BD FACSAria Fusion at the Flow Cytometry & FACS Core Facility (FFCF), Zentrum für Molekulare Biologie der Universität Heidelberg (ZMBH) or FACS Core Facility (dFCCU), Uniklinikum Heidelberg, respectively.

### Genomic DNA extraction from mammalian cell culture

After FACS HEK293T, N2a cells were collected in 500 µl DMEM GlutaMAX supplemented with 10% FBS. hiPSCs were collected in 500 µl StemMACS PSC-Brew XF medium supplemented with 2.5 µM Rock Inhibitor. Cells were collected by centrifugation at 20,000 × g for 2 min, washed with 500 µl D-PBS, and lysed in mammalian gDNA lysis buffer (10 mM Tris–HCl, 0.05% SDS, pH 8, 800 units proteinase K (NEB)) for 2 h at 37 °C to extract genomic DNA. Afterwards, proteinase K was inactivated for 30 min at 80 °C. Cells transfected without fluorophore plasmid were washed once 72 h after transfection, and then lysed in mammalian gDNA lysis buffer, as described above.

### Microinjection of mouse zygotes

Injections were performed as previously described^84^, with minor modifications. In brief, the injection mix contained adenine base editor mRNA (100 ng/μl) and sgRNAs (50 ng/μl each; 12.5 ng/µl each, for pooled sgRNA injections) in injection buffer (10 mM Tris–HCl, 0.25 mM EDTA, pH 7.4) to a final volume of 50 μl with molecular biology-grade water. To prevent clogging of the cannula, the injection mix was filtered shortly before microinjection using a Corning Costar Spin-X 0.22 μm centrifuge tube filter. Zygotes were collected from timed matings and cultured *in vitro* in M2 medium during the injection. Injection into the cytoplasm of zygote-stage embryos was conducted by carefully inserting the injection needle at the equatorial level into the cytoplasm of each embryo, delivering a volume of 1–2 picolitres. Subsequently, the embryos were placed in preincubated M16 medium to select lysed and intact embryos. Embryos were transferred on the same day via oviduct transfer to 0.5-day pseudopregnant foster mothers to increase the overall implantation (nidation) rates.

### Genomic DNA extraction from mouse samples

Embryos were collected at embryonic day 14 (E14) from surrogate mothers to ensure sufficient tissue for genomic DNA isolation and downstream amplicon sequencing. For DNA extraction, each embryo was sectioned into smaller pieces, and genomic DNA was isolated using the DNeasy Blood & Tissue Kit (Qiagen) following the manufacturer’s protocol, with minor modifications. All reagents were applied at double the recommended volume since the starting material exceeded 10 mg per embryo. Proteinase K from New England Biolabs (NEB) was utilised instead of the kit-supplied enzyme.

For genotyping of founder mice, ear biopsies were obtained from the Interfakultäre Biomedizinische Forschungseinrichtung of Heidelberg University. Genomic DNA was extracted from ear biopsy samples using a lysis buffer consisting of DirectPCR Lysis Reagent (Mouse Tail) (Viagen Biotech) and 0.25 µg/µl Proteinase K (AppliChem). For lysis, each sample was incubated in 100–150 µl of this lysis buffer at 55 °C with vigorous shaking for a minimum of 4–5 h, followed by inactivation of Proteinase K at 85 °C for 45 min. The lysate is subsequently used directly for PCR.

### Off-target analysis in mouse embryos

The top five predicted off-target sites for each target locus were identified using ACEofBASEs^56^ as described under sgRNA design (Supplementary Table 7–10). ACEofBASEs provides links to Ensembl gene IDs; therefore, the locus information for these sites was extracted from Ensembl. Importantly, the column “distance” in the off-target list was used when exporting Ensembl sequence information. In brief, when navigating the ENSEMBL genome browser, to export the locus information, ensure the value of distance and an additional 1 kb is added to the “5' Flanking sequence” and “3' Flanking sequence”. Primers were designed (Primer3 2.3.7) to flank the predicted off-target loci. For cumulative editing, all reads containing A-to-G edits in the spacer sequence were added together.

### Targeted deep sequencing and data analysis

Samples were prepared for Amplicon-EZ-based NGS analysis (GeneWiz/Azenta Life Sciences) as previously described^56^. All locus-specific primers contained the 5’ partial Illumina adapter sequences (Fwd: 5’-ACACTCTTTCCCTACACGACGCTCTTCCGATCT-3’, Rev: 5’-GACTGGAGTTCAGACGTGTGCTCTTCCGATCT-3’), and are listed in Supplementary Table 5. Typically, samples corresponding to different target loci from each biological replicate were pooled into a single sample. The pooled NGS data were demultiplexed by mapping to the respective reference sequences later.

For a higher level of sample multiplexing, a sequencing library with indexing primers that include unique i5 and i7 indexes, as well as P5 and P7 adapter sequences, was established. In brief, following the preparation of PCR amplicons with locus-specific primers as described above (PCR1), a second round of PCR was performed on these amplicons to barcode each sample, thereby allowing for the pooling of samples from both the same and different target sites (PCR2). The primer pairs for the second PCR contain complementary regions to the partial Illumina adapters along with the unique i5 and i7 index combinations (Supplementary Table 6). For multiplexing, PCR1 samples were amplified using Q5 Hot Start DNA polymerase (NEB) for 27 cycles, validated by agarose gel electrophoresis of a subsample, and diluted between 1:5 and 1:20 based on gel band density. PCR2 was then set up at a volume of 20 µl, containing 1 µl of the diluted PCR1 product, 0.5 µM of each forward (i5) and reverse primer (i7) to create a combinatorial dual-index library, and Phusion U Green Multiplex PCR Master Mix (Thermo Fisher Scientific); it was amplified for an additional 13 cycles. Individual PCR2 amplification was validated by agarose gel electrophoresis of a subsample, and 24 PCR2 samples were pooled, run on a 1% agarose gel, with specific bands excised and cleaned up using the Monarch DNA Gel Extraction Kit (NEB), measured by Qubit 4 Fluorometer (Thermo) using the Qubit dsDNA BR Assay Kit, and pooled to equimolarity totalling 1 µg DNA. Samples were sequenced by GeneWiz (Azenta Life Sciences) using the Sequencing only service with a 5% PhiX spike-in on an Illumina MiSeq with 2 × 250 bp, paired-end sequencing, at a depth of approximately 10 million reads.

NGS data (in fastq.gz format) were processed and analysed using CRISPResso2 version 2.2.11^85^. In brief, two types of analyses were run in batch mode. First, all samples were analysed in base editing mode with the following custom input parameters: –min_average_read_quality, 30; –quantification_window_size, 20; –quantification_window_center, -10; –base_editor_output. Downstream analysis was conducted using R version 4.2.1 in R Studio (packages: tidyverse, ggplot, ggpubr, cowplot), with data used to plot Adenines across the protospacer sourced from the “Nucleotide_percentage_summary_around_sgRNA.txt” output file. “Precision” (Supplementary Fig. 1) was calculated as previously described^24^ by dividing the second highest adenine-based editing frequency by the highest (A5 or A6); for this purpose, the indel frequency was extracted from the “CRISPRessoBatch_quantification_of_editing_frequency.txt” file and calculated as previously described^56^.

Second, to quantify precise gene editing outcomes, the HDR mode was employed with the following custom input parameters: –min_average_read_quality, 30; –expected_hdr_amplicon_seq; –quantification_window_coordinates, “spacer-7nt (upstream)” – “spacer + PAM sequence (+ 3nt)”; –discard_indel_reads, TRUE. Downstream analysis was conducted using R, as described above, with data sourced from the “CRISPRessoBatch_quantification_of_editing_frequency.txt” file, with slight modifications following^86^. In short, for each sample’s HDR amplicon, values under “Reads_aligned_all_amplicons”, “Reads_aligned”,“Unmodified”, “Modified”, and “Discarded” (HDR) were collected; additionally, the values under “Discarded” (REF) from the Reference amplicon were obtained. Specific values were then calculated as follows:

1 $$\begin{aligned} & {\mathrm{Specified}}\;{\mathrm{mutation}},{\mathrm{incl}}.\;{\mathrm{bystander}}\;{\mathrm{edits}}\left( \% \right) \\ & \quad = \frac{{``{\mathrm{Reads}}\_{\mathrm{aligned}}{{\mathrm{''}}}}}{{``{\mathrm{Reads}}\_{\mathrm{aligned}}\_{\mathrm{all}}\_{\mathrm{amplicons}}{\mathrm{''}}}} \times 100 \\ \end{aligned}$$

2 $$\begin{aligned} & {\mathrm{Specified}}\;{\mathrm{mutation}}\;{\mathrm{without}}\;{\mathrm{bystander}}\;{\mathrm{edits}}\left( \% \right) \\ & \quad = \frac{{\text{``Unmodified{\mathrm{''}}}}}{{{\mathrm{``Reads}}\_{\mathrm{aligned}}\_{\mathrm{all}}\_{\text{amplicons{\mathrm{''}}}}}} \times 100 \\ \end{aligned}$$

3 $${\mathrm{Purity}}\left( \% \right) = \frac{{{\mathrm{``Unmodified}}{\mathrm{''}}}}{{{\mathrm{``Reads}}\_{\mathrm{aligned}}{\mathrm{''}}}} \times 100$$

4 $${\mathrm{Indels}}\left( \% \right) = \frac{{{\mathrm{``Discarded}}{\mathrm{''}}\left( {{\mathrm{HDR}}} \right) + {\mathrm{``Discarded}}{\mathrm{''}}\left( {{\mathrm{REF}}} \right)}}{{{\mathrm{``Reads}}\_{\mathrm{aligned}}\_{\mathrm{all}}\_{\mathrm{amplicons}}{\mathrm{''}}}} \times 100$$

### Statistics and graphics

All data reported in the text are presented as mean ± standard deviation (SD). Where appropriate (n ≥ 20), we also reported the median (Q2) and interquartile range (IQR) between the first quartile (Q1) and the third quartile (Q3). Statistical comparisons were conducted using two-tailed Welch’s *t*-test, as specified in the figure legends. Details on data reporting, including sample sizes and the statistical methods employed in each experiment, are provided in the figure legends. Data demonstrating statistical significance are depicted in the figures. Analysis and graphical data visualisation were performed in R version 4.2.1 with the Tidyverse, ggplot2, ggpubr, and cowplot packages^87–90^. Figures were assembled in Adobe Illustrator, with some icons and schemes imported from Biorender.

## Supplementary Information

Below is the link to the electronic supplementary material.

Supplementary Material 1

Supplementary Material 2

## Data Availability

Oligonucleotide and sgRNA sequences used in this study are listed in Supplementary Tables 2, 3, 5, and 6. All raw NGS sequencing data generated in this study were in the NCBI Sequence Read Archive (SRA) database as a BioProject with the accession number PRJNA1291991. Plasmids encoding pCMV_ABE9-SpRY (/#242976) and pEF1α_ABE9-SpRY (#242977) are available at Addgene. Any other data and reagents will be made available upon reasonable request.
