# Supplementary material for: ABE9 fused to SpRY Cas9 nickase enables precise generation of bystander free mouse models
Source: Sci Rep. 2026 Feb 20;16:7463. doi: 10.1038/s41598-026-40642-z (PMC12929607; doi:10.1038/s41598-026-40642-z)
Supplement: Supplementary file 1 — Supplementary Material 1 [file 41598_2026_40642_MOESM1_ESM.pdf]

SUPPLEMENTARY FIGURES

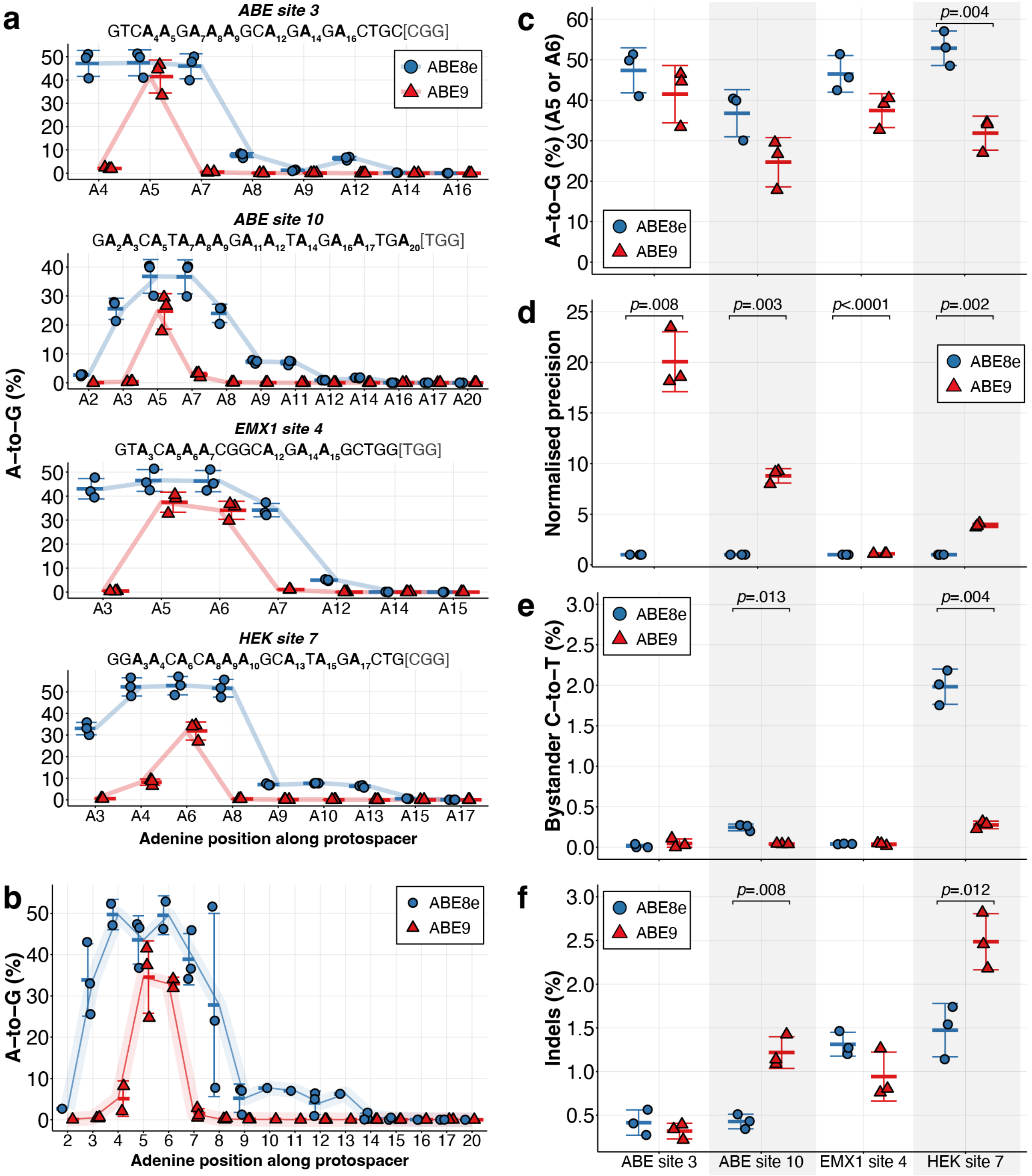

Supplementary Figure 1 | ABE9's precision outweighs the higher A-to-G efficiency of ABE8e in human cells. (**a**) Editing efficiencies of ABE8e and ABE9 across four endogenous target sites. (**b**) Summary plot of editing window across the four loci. (**c**) A-to-G editing frequency at adenine positions A5 or A6 (the highest is considered) at the four target sites. (**d**) Normalised precision was calculated as described by Chen et al. (*Nat Chem Biol*, 2023). For each target site, we first computed a precision ratio as the highest A-to-G editing frequency across editable adenines divided by the second-highest frequency (peak/runner-up). This ratio was then normalised to the corresponding ABE8e reference value (ABE8e used for standardisation). (**e**) Unwanted C-to-T editing frequency measured across the protospacer sequence. (**f**) Indel frequency across the target sites. Individual data points for three independent biological replicates are shown. Error bars indicate standard deviation (SD); bounds were capped at 0% and 100% where the mean ± SD exceeded the valid range. Statistical significance was determined by two-tailed Welch's *t*-test.

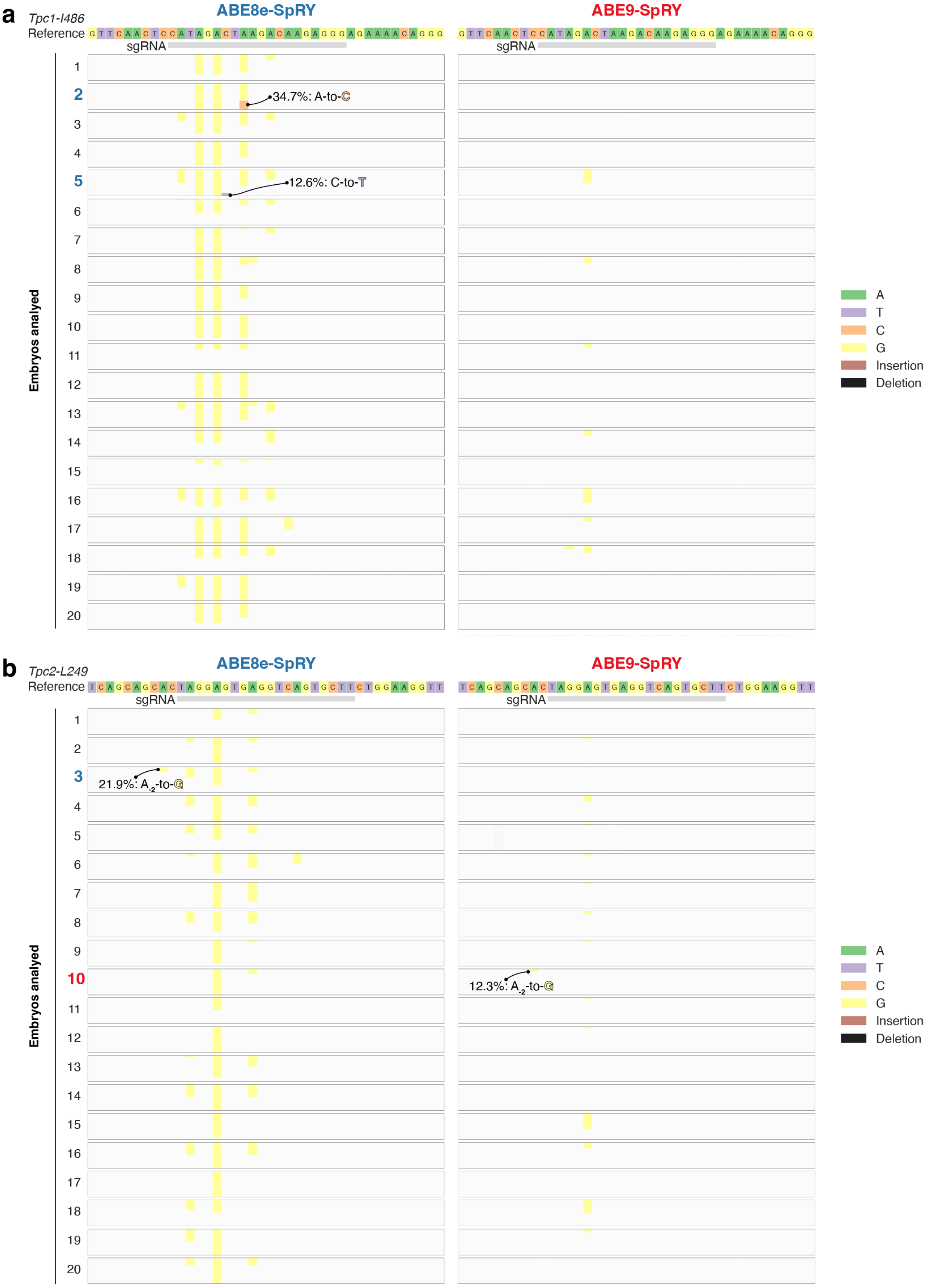

Supplementary Figure 2 | Undesired on-target editing with ABE8e-SpRY and ABE9-SpRY at the *Tpc1^{I486}* (a) and *Tpc2^{L249}* (b) loci. Graphs show the base composition in Crispresso2 plots of NGS analysis, highlighting, in colour, any modifications in edited embryos compared to the reference sequence 10 bp downstream and upstream of the sgRNA target site. The vertical size corresponds to the frequency of the modification.

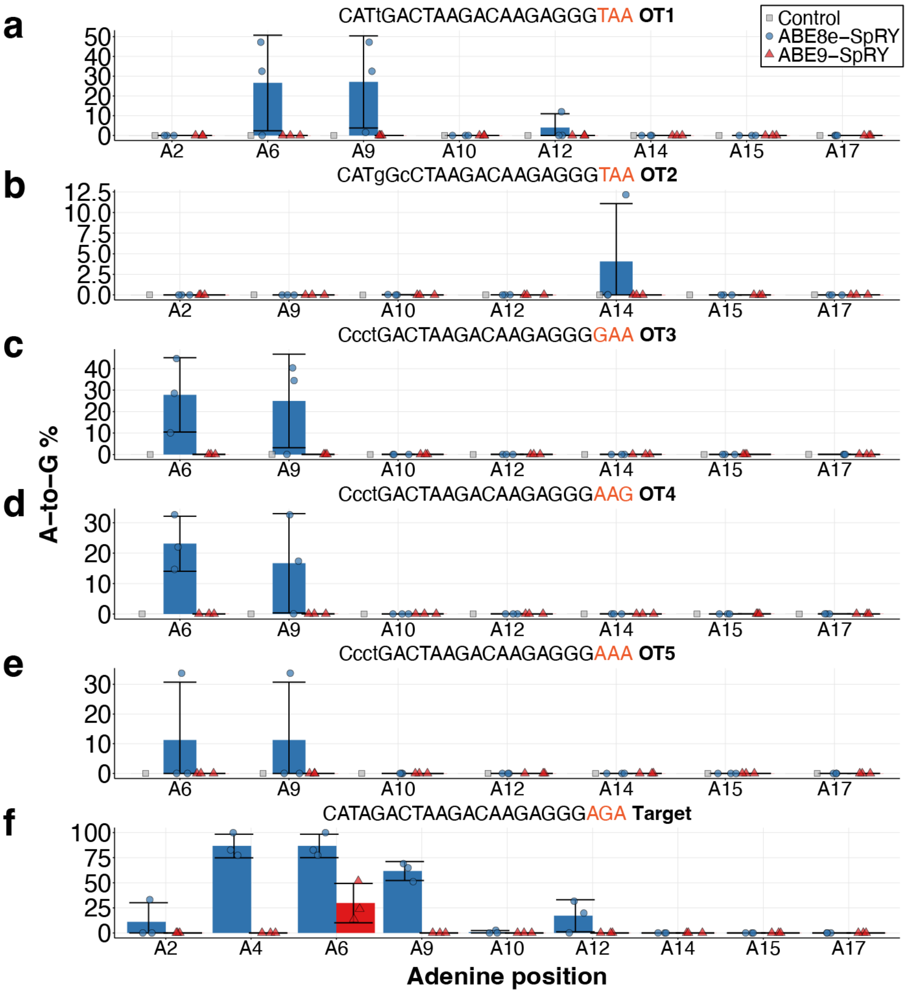

Supplementary Figure 3 | ***Tpc1**[I486]* **off-target analysis.** (**a-e**) A-to-G frequency analysis of the top five predicted off-target sites across all adenines within the protospacer sequence. Mismatches compared to the on-target sequence (**f**) are shown by lowercase letters, while the PAM sequence is highlighted in orange. (**f**) Replotting of the on-target A-to-G editing efficiency for the embryos used for off-target evaluation (Fig. 3c). Individual data points for three independent biological replicates are shown. Error bars indicate standard deviation (SD); bounds were capped at 0% and 100% where the mean ± SD exceeded the valid range. Control, gDNA from ear biopsy samples of adult wild-type mice.

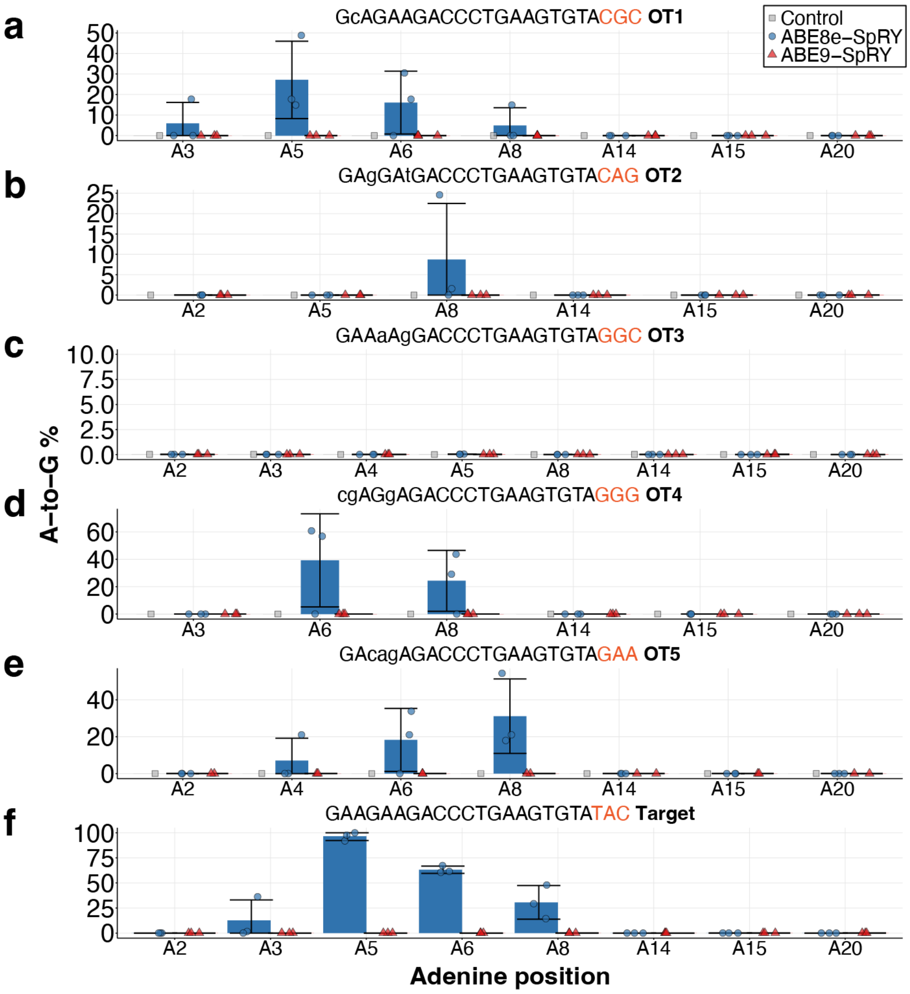

Supplementary Figure 4 | *Tpc2*^{K188} off-target analysis. (a-e) A-to-G frequency analysis of the top five predicted off-target sites across all adenines within the protospacer sequence. Mismatches compared to the on-target sequence (**f**) are shown by lowercase letters, while the PAM sequence is highlighted in orange. (**f**) Replotting of the on-target A-to-G editing efficiency for the embryos used for off-target evaluation (Fig. 3c). Individual data points for three independent biological replicates are shown. Error bars indicate standard deviation (SD); bounds were capped at 0% and 100% where the mean ± SD exceeded the valid range. Control, gDNA from ear biopsy samples of adult wild-type mice.

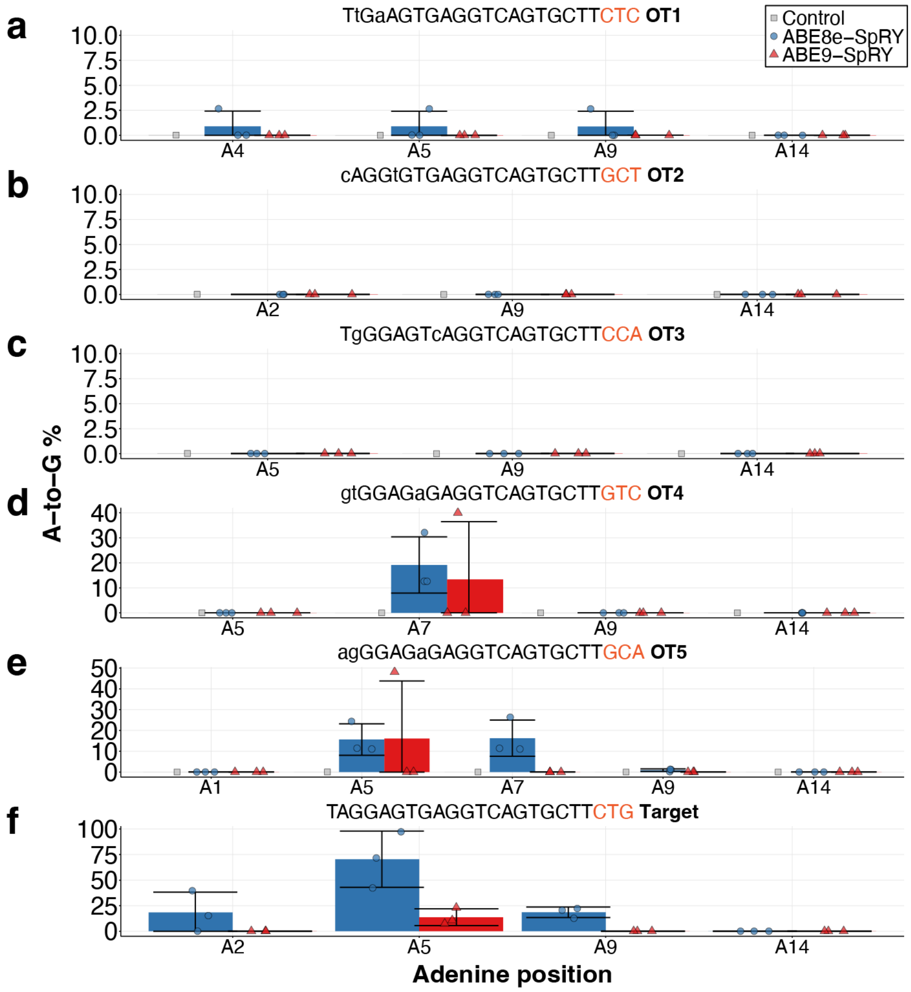

Supplementary Figure 5 | ***Tpc2*[L249] off-target analysis.** (**a-e**) A-to-G frequency analysis of the top five predicted off-target sites across all adenines within the protospacer sequence. Mismatches compared to the on-target sequence (**f**) are shown by lowercase letters, while the PAM sequence is highlighted in orange. (**f**) Replotting of the on-target A-to-G editing efficiency for the embryos used for off-target evaluation (Fig. 3c). Individual data points for three independent biological replicates are shown. Error bars indicate standard deviation (SD); bounds were capped at 0% and 100% where the mean ± SD exceeded the valid range. Control, gDNA from ear biopsy samples of adult wild-type mice.

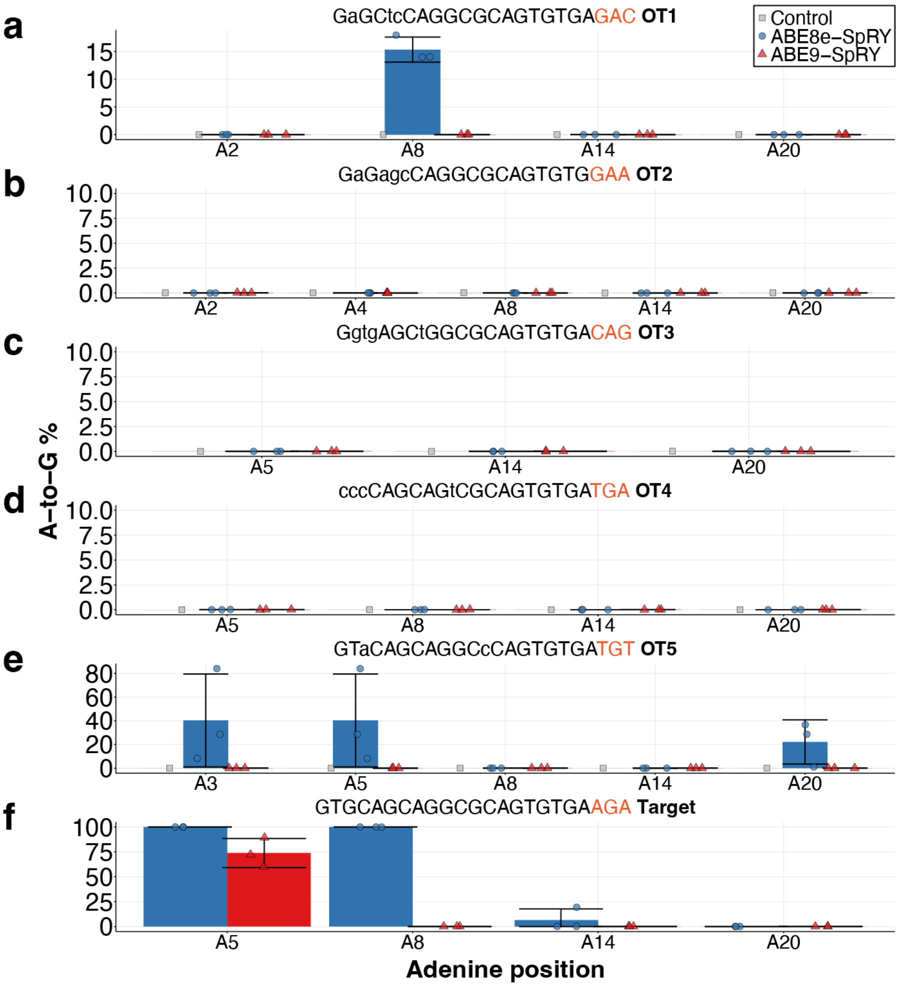

Supplementary Figure 6 | ***Trpm4*^{L903} off-target analysis.** (**a-e**) A-to-G frequency analysis of the top five predicted off-target sites across all adenines within the protospacer sequence. Mismatches compared to the on-target sequence (**f**) are shown by lowercase letters, while the PAM sequence is highlighted in orange. (**f**) Replotting of the on-target A-to-G editing efficiency for the embryos used for off-target evaluation (Fig. 3c). Individual data points for three independent biological replicates are shown. Error bars indicate standard deviation (SD); bounds were capped at 0% and 100% where the mean ± SD exceeded the valid range. Control, gDNA from ear biopsy samples of adult wild-type mice.

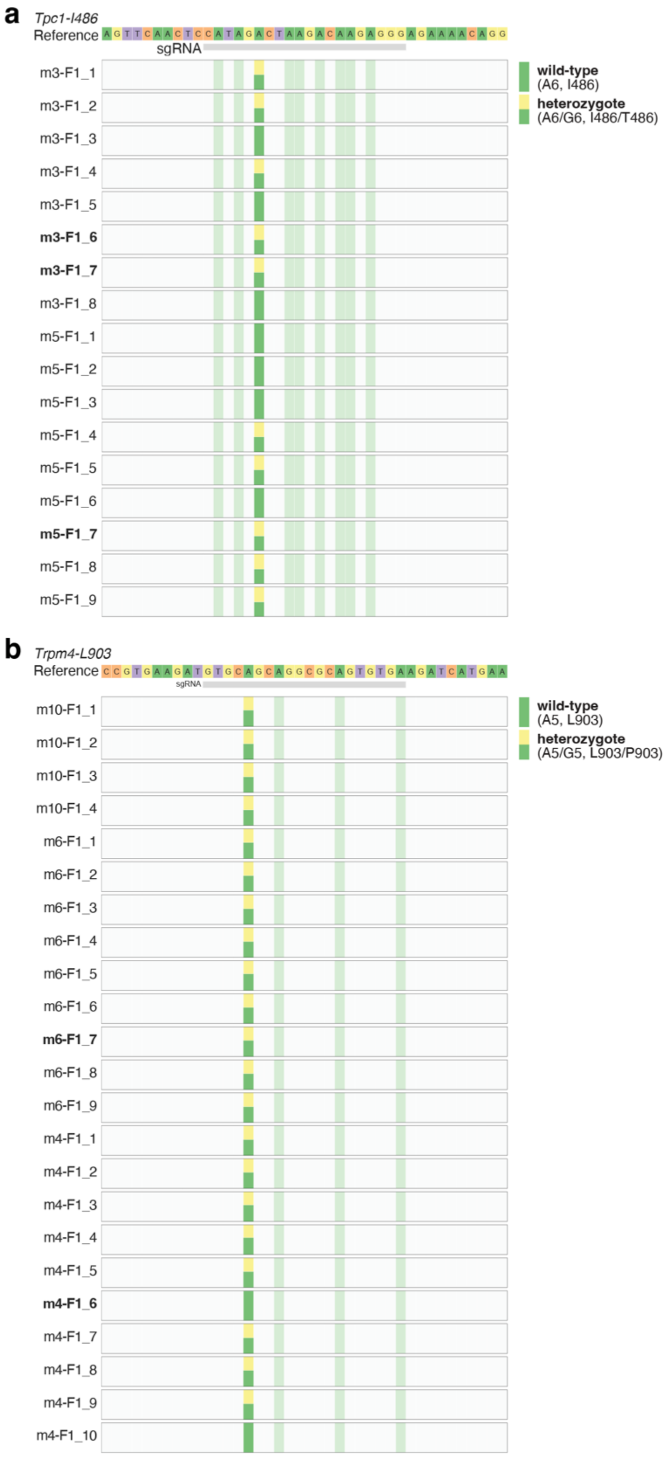

Supplementary Figure 7 | Analysis of F1 transmission from ABE9-SpRY edited sites at the *Tpc1*^{I486} (a) and *Trpm4*^{L903} (b) loci. Graphs show the base composition in Crispresso2 plots of NGS analysis, highlighting, in colour, any modifications in edited adult mice from ear biopsies compared to the reference sequence 10 bp downstream and upstream of the sgRNA target site. The vertical size corresponds to the frequency of the modification. A total for 17 (**a**) and 21 (**b**), adult mice were analysed to identify heterozygous carriers of the *Tpc1*^{I486T} and *Trpm4*^{L903P}, respectively.

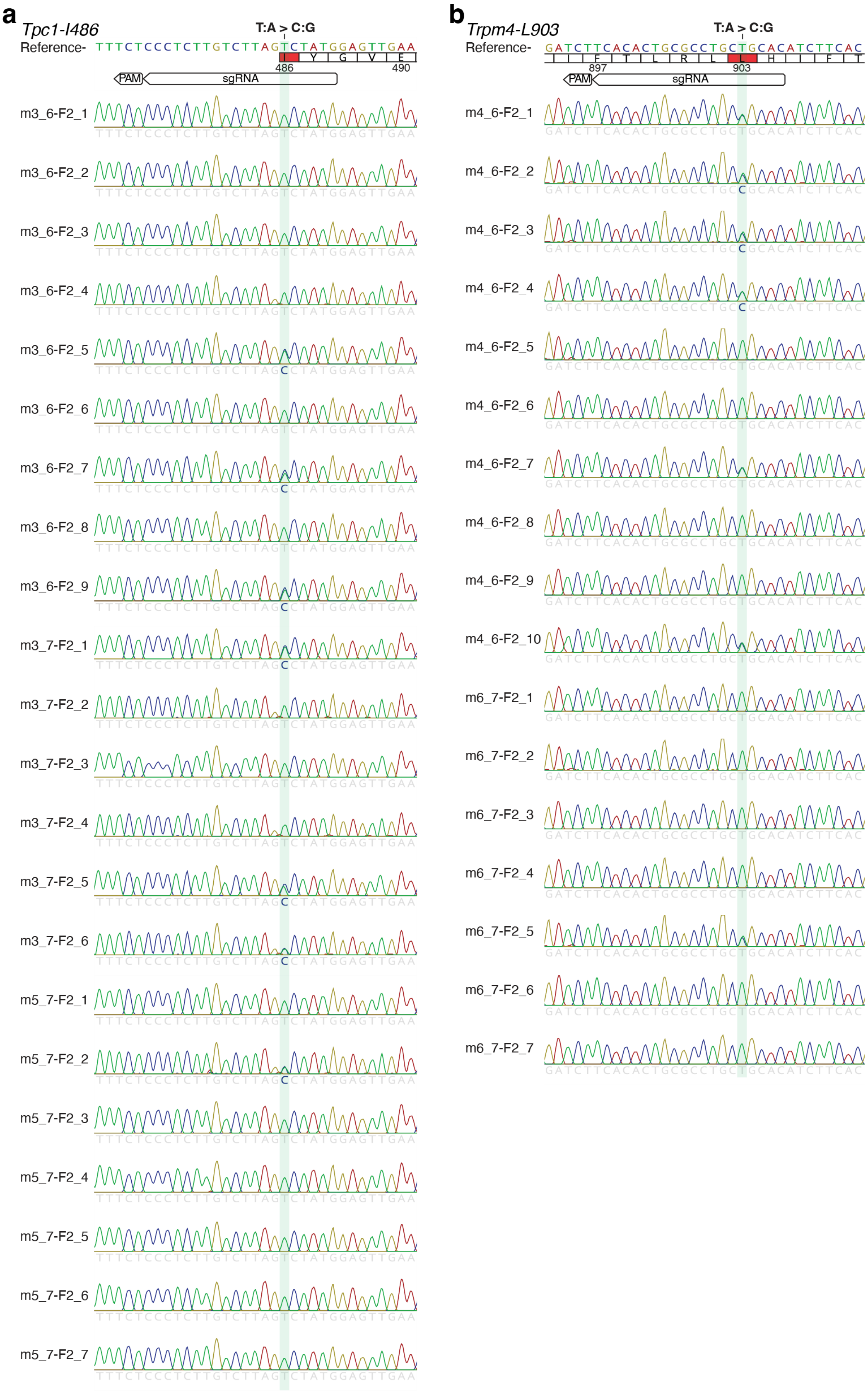

Supplementary Figure 8 | Analysis of F2 transmission from ABE9-SpRY edited sites at the *Tpc1^{I486}* (a) and *Tpc2^{L249}* (b) loci. Sanger sequencing reads for 22 and 17 F2 mouse ear biopsies were analysed for heterozygous carrier status of the *Tpc1^{I486T}* and *Trpm4^{L903P}* mutations, respectively. Note: for both targets, the sgRNA targets the complementary strand. Green shade highlights the mutated base.

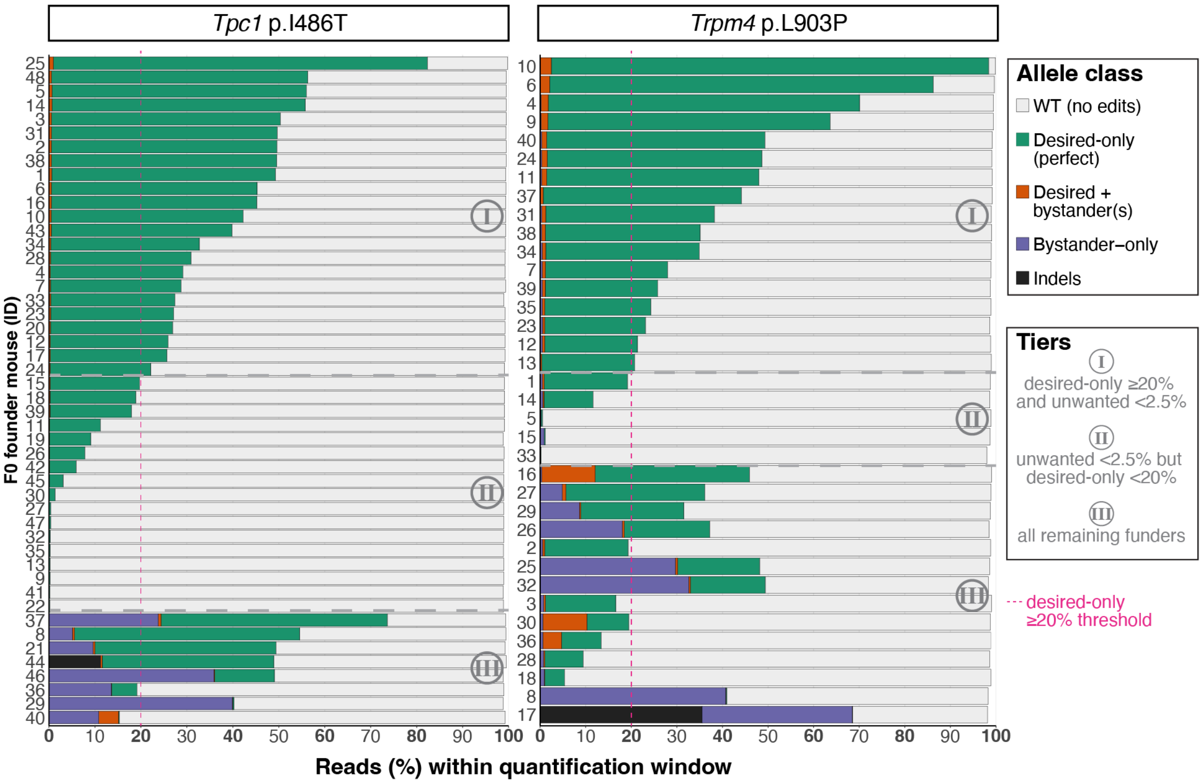

Supplementary Figure 9 | Per-founder allele-class composition reveals read-level co-occurrence of desired and bystander edits in F0 founders. Amplicon sequencing reads were classified into allele classes using CRISPResso2 within the predefined quantification window (spacer −7 nt upstream to spacer + PAM (+3 nt); see Methods). For each F0 founder, stacked bars indicate the proportion of reads that were WT, desired-only ("perfect"; desired substitution without additional edits within the window), desired + bystander(s), bystander-only, or indel-containing. "Unwanted" outcomes were defined as the combined fraction of desired + bystander(s), bystander-only, and indels. Founders are ordered within each locus by tier and then by desired-only fraction (descending). Tier I ("strong clean allele-carrying") founders meet the criteria desired-only ≥20% and unwanted ≤2.5%; Tier II founders exhibit unwanted ≤2.5% but desired-only <20%; Tier III comprises all other founders. The dotted line indicates the 20% desired-only threshold, and dashed lines separate tiers.

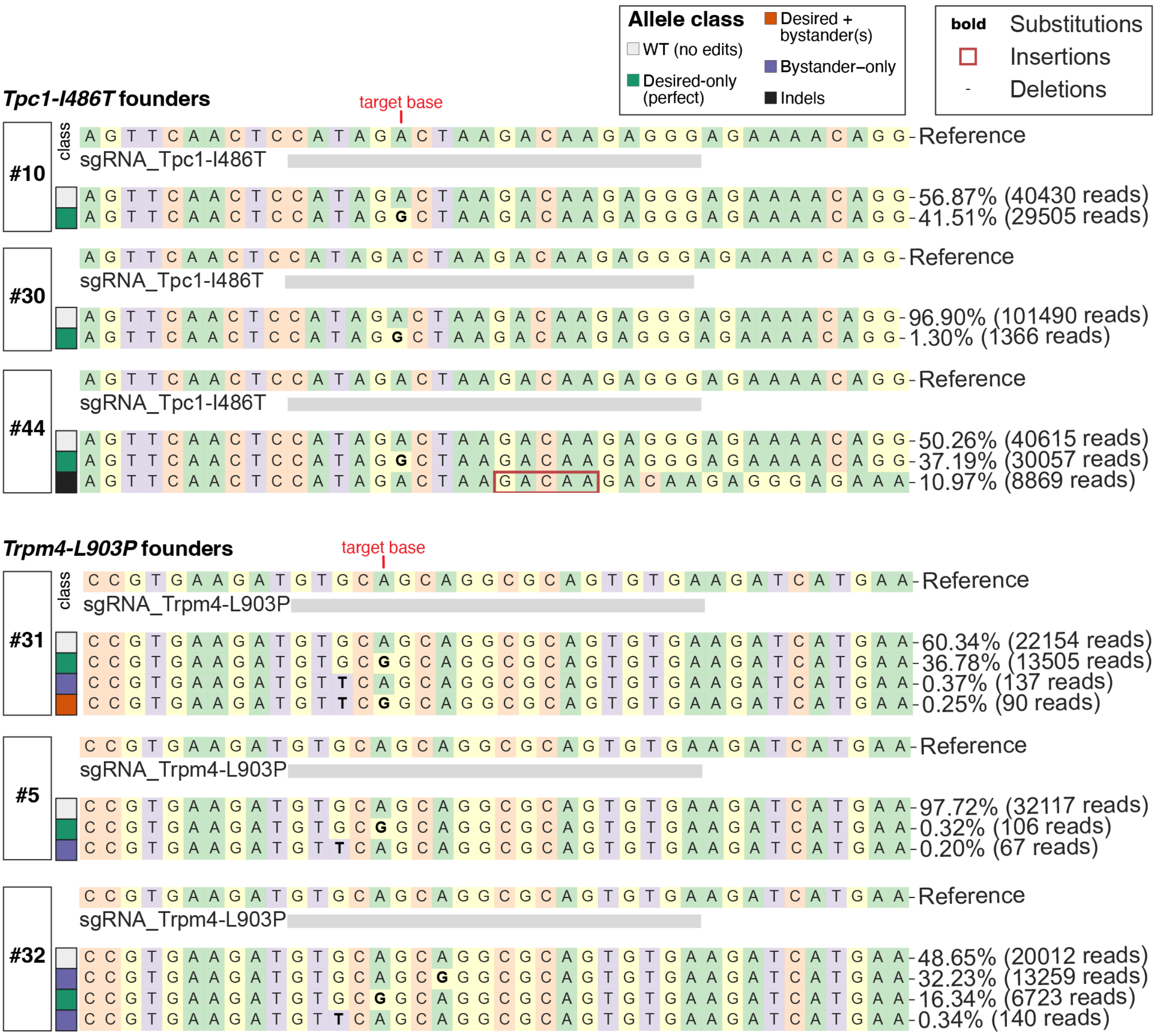

Supplementary Figure 10 | Representative allele spectra from CRISPResso2 allele frequency tables for F0 founders in each tier. CRISPResso2 allele frequency outputs are shown for one representative F0 founder per tier at each locus (*Tpc1-I486* and *Trpm4-L903*). For each line, founders were first assigned to Tier I–III as defined in Supplementary Fig. S9 and then ordered within each tier by desired-only ("perfect") fraction; the displayed example corresponds to the median-ranked founder (rank ⌈n/2⌉) within that tier. Allele frequencies are derived from amplicon reads within the predefined quantification window and illustrate the co-occurrence of the desired substitution with bystander edits and/or indels at the read level.

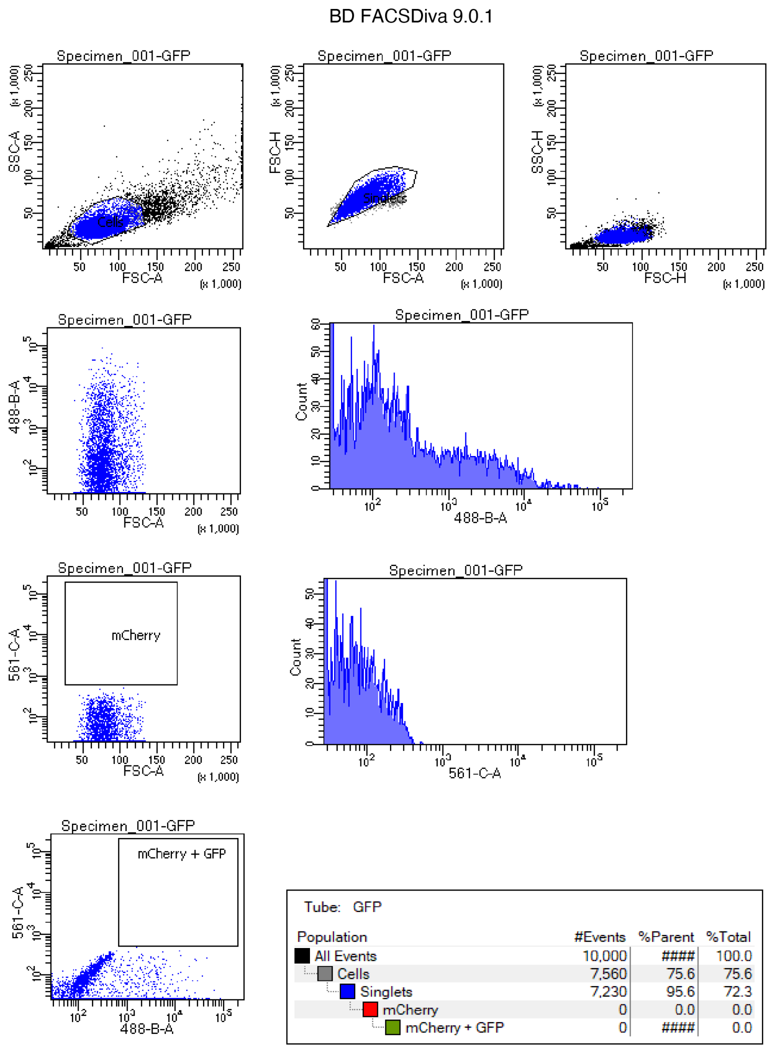

Population	#Events	%Parent	%Total
All Events	10,000	####	100.0
Cells	7,560	75.6	75.6
Singlets	7,230	95.6	72.3
mCherry	0	0.0	0.0
mCherry + GFP	0	####	0.0

Supplementary Figure 11 | Representative FACS plots and gating strategy for XMAS-TREE data in hiPSCs. Data show GFP-control-only transfected wild-type hiPSCs.

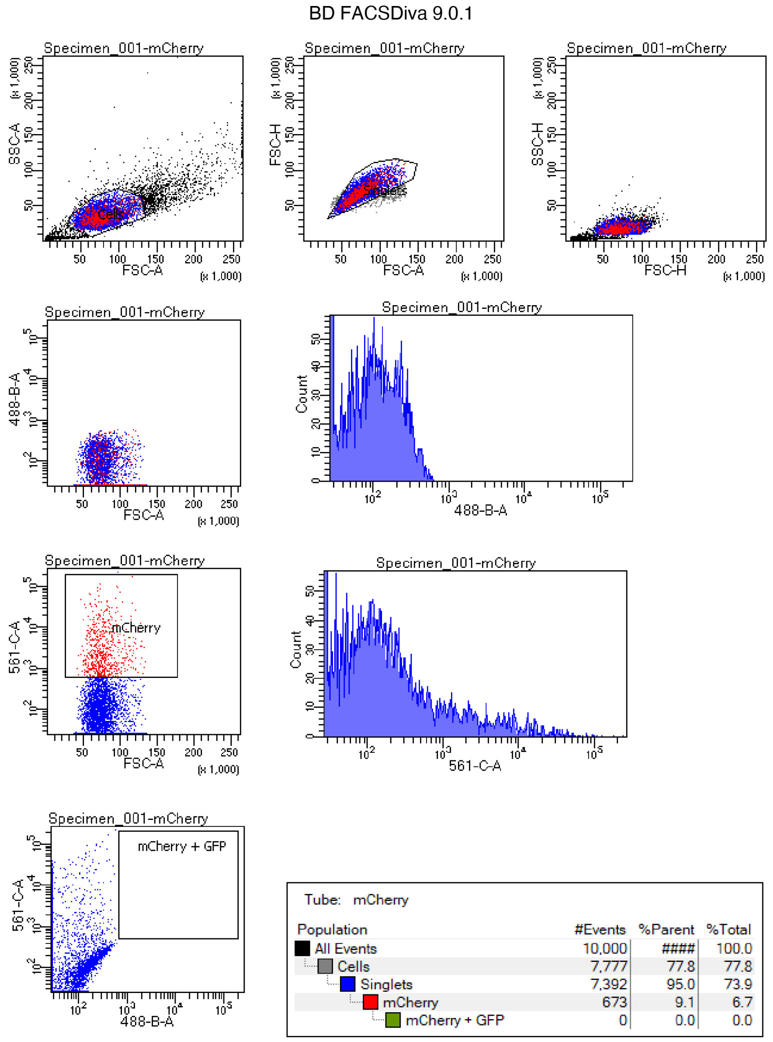

Population	#Events	%Parent	%Total
All Events	10,000	####	100.0
Cells	7,777	77.8	77.8
Singlets	7,392	95.0	73.9
mCherry	673	9.1	6.7
mCherry + GFP	0	0.0	0.0

Supplementary Figure 12 | Representative FACS plots and gating strategy for XMAS-TREE data in hiPSCs. Data show mCherry-control-only transfected wild-type hiPSCs.

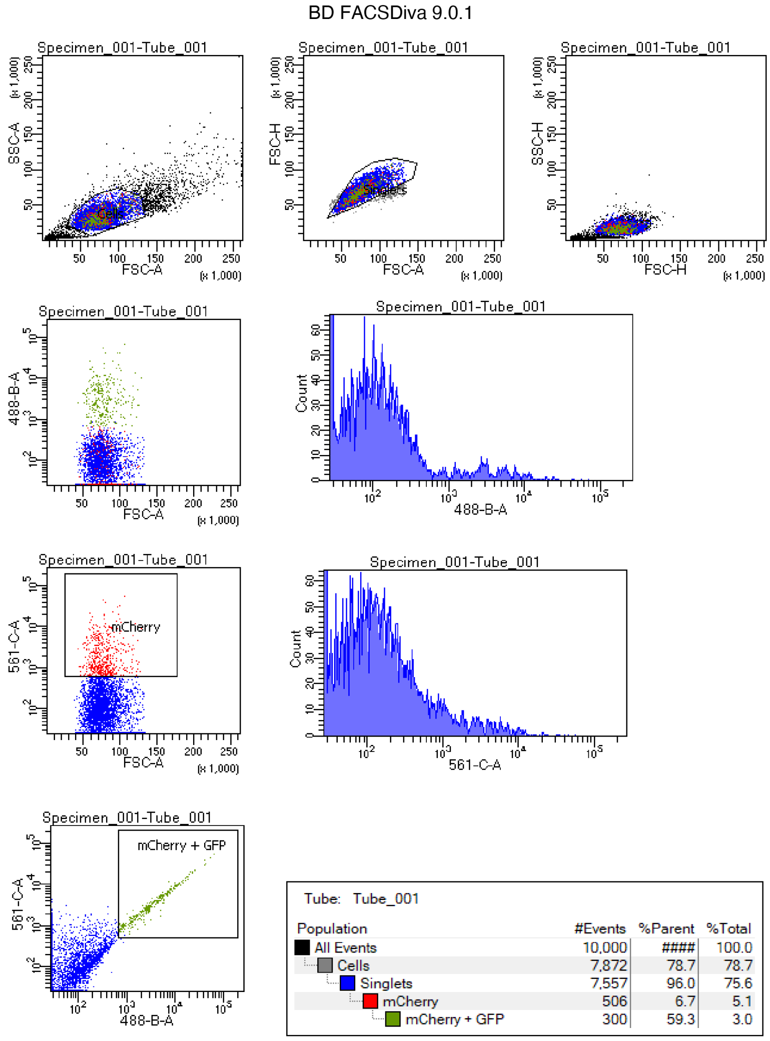

Tube: Tube_001

Population	#Events	%Parent	%Total
All Events	10,000	####	100.0
Cells	7,872	78.7	78.7
Singlets	7,557	96.0	75.6
mCherry	506	6.7	5.1
mCherry + GFP	300	59.3	3.0

Supplementary Figure 13 | Representative FACS plots and gating strategy for XMAS-TREE data in hiPSCs. Data show a replicate of hiPSCs transfected with ABE8e-SpRY and sgRNA1.

SUPPLEMENTARY TABLE CAPTIONS

Supplementary Table 1 | Key resources. Key reagents, plasmids, cell lines, genes, and software used in this study, including source information and identifiers where available.

Supplementary Table 2 | Oligonucleotides to clone sgRNAs used in this study. Top and bottom oligo sequences provided in 5' to 3' orientation for all sgRNAs used as plasmids in this study, along with the oligos for the *Sp*Cas9 and *Sa*Cas9 sgRNA scaffolds.

Supplementary Table 3 | sgRNA sequences. sgRNA target site (protospacer) sequences corresponding to the sgRNA spacer sequence with the PAM shown in brackets are provided in 5' to 3' orientation for all sgRNAs used in this study. This includes information on the mode of use (as standard SpCas9 or SaCas9 plasmid, XMAX-TREE plasmid, or AltR-sgRNA as an RNA molecule) along with source details.

Supplementary Table 4 | Oligonucleotides for HiFi assembly cloning. Forward and reverse primer sequences (5′ to 3′) used to amplify DNA fragments for HiFi assembly cloning of plasmids generated in this study. Template DNA source and expected amplicon size (bp) are provided for each fragment.

Supplementary Table 5 | Target amplicon oligonucleotides used for PCR1. Locus-specific primer pairs (5′ to 3′) used in the first PCR (PCR1) to amplify genomic regions for amplicon deep sequencing of on-target and/or selected off-target sites.

Supplementary Table 6 | Barcoding oligonucleotides used for PCR2. Primer sequences (5′ to 3′) used in the second PCR (PCR2) to append sample-specific barcodes and sequencing adapters for multiplexed amplicon sequencing.

Supplementary Table 7 | ACEofBASEs predicted off-target sites for the *Tpc1-I486* sgRNA. ACEofBASEs *in silico* off-target prediction output for the *Tpc1-I486* sgRNA. This table lists the predicted Cas9-dependent off-target sites and associated alignment/ranking information used to define the candidate off-target panel quantified in embryos.

Supplementary Table 8 | ACEofBASEs predicted off-target sites for the *Tpc2-K188* sgRNA. ACEofBASEs *in silico* off-target prediction output for the *Tpc2-K188* sgRNA. Predicted Cas9-dependent off-target sites and associated alignment/ranking information are provided for off-target panel selection.

Supplementary Table 9 | ACEofBASEs predicted off-target sites for the *Tpc2-L249* sgRNA. ACEofBASEs *in silico* off-target prediction output for the *Tpc2-L249* sgRNA. Predicted Cas9-dependent off-target sites and associated alignment/ranking information are provided for off-target panel selection.

Supplementary Table 10 | ACEofBASEs predicted off-target sites for the *Trpm4-L903* sgRNA. ACEofBASEs *in silico* off-target prediction output for the *Trpm4-L903* sgRNA. Predicted Cas9-dependent off-target sites and associated alignment/ranking information are provided for off-target panel selection.

Supplementary Table 11 | Raw cumulative A-to-G editing measurements at off-target sites. Raw per-replicate cumulative A-to-G (%) values for each base-editing condition across loci and sites (including off-target sites). "Cumulative" values represent the summed A-to-G editing across adenines in the protospacer/quantification window as defined in Methods.

Supplementary Table 12 | XMAS-TREE FACS enrichment metrics in hiPSCs. Flow cytometry quantification of XMAS-TREE reporter activation following transfection with the indicated editor and sgRNA. The table reports (i) the percentage of mCherry+ cells in the total population and (ii) the percentage of mCherry+GFP+ cells within the mCherry+ population, with mean and SD values as indicated.
